# Genome sequencing and functional analysis of a multipurpose medicinal herb *Tinospora cordifolia* (Giloy)

**DOI:** 10.1038/s41598-024-53176-z

**Published:** 2024-02-02

**Authors:** Shruti Mahajan, Abhisek Chakraborty, Manohar S. Bisht, Titas Sil, Vineet K. Sharma

**Affiliations:** MetaBioSys Group, Department of Biological Sciences, Indian Institute of Science Education and Research Bhopal, Bhopal, Madhya Pradesh 462066 India

**Keywords:** Computational biology and bioinformatics, Evolution, Plant sciences

## Abstract

*Tinospora cordifolia* (Willd.) Hook.f. & Thomson, also known as Giloy, is among the most important medicinal plants that have numerous therapeutic applications in human health due to the production of a diverse array of secondary metabolites. To gain genomic insights into the medicinal properties of *T. cordifolia*, the genome sequencing was carried out using 10× Genomics linked read and Nanopore long-read technologies. The draft genome assembly of *T. cordifolia* was comprised of 1.01 Gbp, which is the genome sequenced from the plant family Menispermaceae*.* We also performed the genome size estimation for *T. cordifolia,* which was found to be 1.13 Gbp. The deep sequencing of transcriptome from the leaf tissue was also performed. The genome and transcriptome assemblies were used to construct the gene set, resulting in 17,245 coding gene sequences. Further, the phylogenetic position of *T. cordifolia* was also positioned as basal eudicot by constructing a genome-wide phylogenetic tree using multiple species. Further, a comprehensive comparative evolutionary analysis of gene families contraction/expansion and multiple signatures of adaptive evolution was performed. The genes involved in benzyl iso-quinoline alkaloid, terpenoid, lignin and flavonoid biosynthesis pathways were found with signatures of adaptive evolution. These evolutionary adaptations in genes provide genomic insights into the presence of diverse medicinal properties of this plant. The genes involved in the common symbiosis signalling pathway associated with endosymbiosis (Arbuscular Mycorrhiza) were found to be adaptively evolved. The genes involved in adventitious root formation, peroxisome biogenesis, biosynthesis of phytohormones, and tolerance against abiotic and biotic stresses were also found to be adaptively evolved in *T. cordifolia.*

## Introduction

*Tinospora cordifolia* is a climbing shrub belonging to the Menispermaceae family that includes more than 400 plant species^1^. The present scientific name of this plant is *Tinospora cordifolia* (Willd.) Hook.f. & Thomson according to the ‘USDA-ARS GRIN Taxonomy’, whereas its scientific name as per the ‘International Plant Names Index’ is *Tinospora cordifolia*. Thus, we have referred to it as *Tinospora cordifolia* in the subsequent text. This plant has gained tremendous therapeutic interest and significance during the COVID-19 pandemic due to its enormous therapeutic potential^2^. It perhaps originated in Africa in the Oligocene epoch (28.57 million years ago) and was spread to Asia in the early Miocene epoch (21.54 million years ago)^3^. It is found in tropical and sub-tropical regions including India, China, Sri Lanka, Bangladesh, Myanmar, Thailand, Malaysia, etc., and is also known as ‘Giloy’, ‘Amrita’, ‘Guduchi’, and ‘heart leaved moonseed’^1^. It is a perennial deciduous dioecious plant with morphological characteristics of twining branches, succulent stem with papery bark, alternatively arranged heart-shaped leaves, aerial roots, and greenish-yellow tiny flowers in the form of racemes inflorescence^1,4^. Being a climber, it needs a supportive plant like *Jatropha curcas* (Jatropha), *Azadirachta indica* (Neem), *Moringa oleifera* (Moringa), etc., for its growth^4^. These supportive plants also play an important role in enhancing the production of various secondary metabolites of *T. cordifolia*^4,5^.

*T. cordifolia* produces an array of secondary metabolites in response to stress conditions, and their concentration also varies based on seasons and its dioecy nature^6^. The chemical constituents of this plant are broadly categorized as alkaloids, terpenoids, phenolics, polysaccharides, steroids, essential oils, aliphatic compounds, etc., which can be obtained from various parts of the plant^7,8^. A study reported that among the two species of *Tinospora* (i.e. *T. cordifolia* and *T. sinensis*), *T. cordifolia* produces three times higher concentration of an alkaloid, berberine, than *T. sinensis,* and thus the former is preferred in therapeutics^9^. Its bioactive compounds have known biological properties such as anti-oxidant, antipyretic, anti-diabetic, anti-inflammatory, anti-microbial, anti-viral, anti-arthritis, anti-osteoporotic, anti-HIV, anti-cancer, hepatoprotective, anti-malarial, immunomodulation, etc.^10,11^. These properties make this species useful in the traditional treatment of several ailments, including fevers, cough, diabetes, general debility, ear pains, jaundice, asthma, heart diseases, burning sensation, bone fracture, urinary problems, chronic diarrhoea, dysentery, leucorrhoea, skin diseases, cancer, helminthiasis, leprosy, and rheumatoid arthritis^12,13^. Further pre-clinical and clinical studies have been carried out to indicate its potential to treat leucopenia induced by breast cancer chemotherapy, hepatic disorders, post-menopausal syndrome, obstructive jaundice, etc.^12^.

These diverse and important therapeutic applications of *T. cordifolia,* due to the presence of secondary metabolites such as alkaloids, terpenoids, lignans, flavonoids, etc., make it a species of broad interest^8^. Further the alkaloids, namely berberine, columbamine, magnoflorine, and other compounds from this plant have proven immunomodulatory activity^14^. The compounds like berberine, cordifoliside C, magnoflorine, tembetarine, cholesterol, melezitose, allopyranose, etc., are reported to possess antimicrobial activity against various bacterial and fungal species^15–20^. These phytochemicals are reported to be effective against chikungunya, dengue, and also against SARS-CoV2 viruses, due to which this herb gained enormous attention and popularity during the COVID-19 pandemic^21–27^. Ten compounds from *T. cordifolia* were predicted as drug candidates against COVID-19 and also found inactive towards the central nervous system, which is essential for a good drug candidate^28^. Not only in humans, it also reduced the mortality rate in chickens infected with the infectious bursal disease virus^29^.

The bioactive compounds in medicinal plants are not only of plant origin but are also produced by the endophytic microorganisms present in them. Plants are associated with a large number of symbiotic microorganisms like endophytic bacteria and fungi. These endophytic microorganisms play roles in the growth, development, and stress tolerance of associated plants^30,31^. One of such microorganisms is arbuscular mycorrhiza fungi (AMF) that forms an association (endosymbiosis) of fungi with the roots of higher plants. AMF have branched structures called arbuscules that facilitate the exchange of minerals between plants and fungi and also improve the nutrient uptake of plants^32^. AMF are known to have impact on secondary metabolite production by increasing the plant biomass or stimulating the biosynthetic pathways for secondary metabolite production. This endosymbiosis occurs in plants through a pathway known as the common symbiosis signalling pathway (CSSP). They have been reported to enhance the production of secondary metabolites, such as alkaloids, terpenoids, phenolics, saponins, etc., in various medicinal plants^33^. The presence of endophytic bacteria and fungi has previously been reported in the leaves, stems, petiole, and roots of this plant^34–37^. These endophytic microorganisms thus potentially contribute to the medicinal properties of this multifaceted plant.

However, despite the widely known medicinal properties of this plant, its genome assembly was still unavailable. A preliminary study reported the transcriptome (482 Mb data) of this species from leaf and stem tissues using 454 GS-FLX pyrosequencing^38^. A recent karyological study reported 2n = 26 as the chromosome number in *T. cordifolia*, which was also supported by earlier studies^39–41^. Thus, to uncover the genomic basis of its medicinal properties and to further explore its therapeutic potential, we carried out the genome sequencing and assembly of *T. cordifolia* using 10× Genomics linked reads and Oxford Nanopore long reads. This is the draft genome assembly of *T. cordifolia*, which is also the genome sequenced so far from the medicinally important genus *Tinospora* and its family^42^. We also carried out a comprehensive deep sequencing and assembly of the leaf transcriptome using Illumina technology. The genome-wide phylogenetic analysis was also carried out for *T. cordifolia* to determine its phylogenetic position. A comprehensive evolutionary comparative analysis including identification of gene families expansion/contraction and signatures of adaptive evolution was performed to gain genomic insights into its medicinal properties.

## Results

### Sampling and sequencing of *T. cordifolia* genome and transcriptome

**Figure 1:**
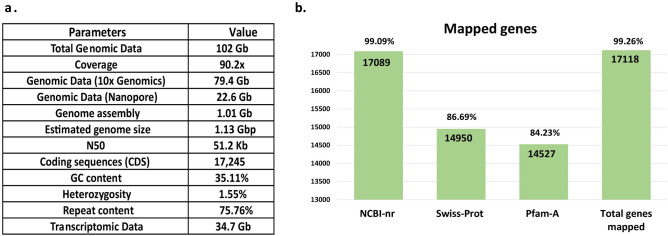
Summary of *T. cordifolia* genome assembly and genes. (**a**) Summary of generated data and genome assembly (**b**) Statistical summary of mapped genes of *T. cordifolia.*

The species was confirmed as *T. cordifolia* with the highest identity (98.32% for ITS and 99.41% for *matK*) with sequences of *T. cordifolia* available at NCBI (nt) database. The total genomics data generated was 102 Gb which comprised 79.4 Gb of 10× genomics linked read and 22.6 Gb of Nanopore read data **(**Fig. 1a and Supplementary Table S1). Additionally, 34.7 Gb of transcriptomic data from the leaf tissue was generated and used with genomic data for the genome assembly and analysis of *T. cordifolia* **(**Fig. 1a and Supplementary Table S2). Since the genome size was not available for this plant, the genome size for *T. cordifolia* was computationally estimated to be 1.13 Gbp. Considering this genome size, the sequenced genomic data provided 90.2 × genome coverage. Further, the *T. cordifolia* genome was found to have high heterozygosity (1.55%) **(**Fig. 1a**)**.

After scaffolding, misassembly rectification, gap-filling, and polishing, the final draft genome assembly of *T. cordifolia* resulted in a 1.01 Gbp assembly size. The assembled genome consisted of 35.11% GC content, 55,140 scaffolds, and N50 of 51.2 Kb **(**Fig. 1a and Supplementary Table S3). The BUSCO completeness was 83% in the final polished *T. cordifolia* genome assembly, and the complete plus fragmented BUSCO score was 94.1%, and the missing BUSCOs was 5.9% (Supplementary Table S4). Similarly, the BUSCO completeness was 60.2% in the final polished *T. cordifolia* genome annotation, and the complete plus fragmented BUSCO score was 80.9%, and the missing BUSCOs was 19.1% (Supplementary Table S4). The de novo transcriptome assembly resulted in a 2,764,154 bp size, and 8208 transcripts were predicted in the assembled transcriptome.

### Annotation of genome and gene set formation

The final polished genome assembly consisted of 1875 repeat families, which were further clustered into 1557 representative repeat family sequences. *T. cordifolia* genome was predicted with 75.75% of repetitive sequences. Among these repetitive sequences, 70.73% were characterized as interspersed repeats comprising 53.29% retroelements (48.39% of LTR repeats), 2.29% DNA transposons, and 15.14% unclassified repeats. The LTR repeats comprised 39.48% Gypsy/DIRS1 and 6.84% Ty1/Copia elements (Supplementary Table S5). Among the non-coding RNAs, 394 hairpin miRNAs, 1306 rRNAs, and 2164 tRNAs were also predicted.

A total of 19,264 coding sequences were obtained in the genome assembly of *T. cordifolia*. Among these coding genes, 17,245 genes were present in the high-confidence coding gene set after filtering. Overall, 17,118 genes (99.26%) could be mapped against publicly available databases **(**Fig. 1b**)**. The functional annotations of these high-confidence coding genes are provided in Supplementary Table S6–S8. A large fraction of these genes was involved in KEGG pathways like ribosome, spliceosome, protein processing in endoplasmic reticulum, etc., and COG categories like signal transduction mechanisms, post translational modification, protein turnover, chaperones, carbohydrate transport and metabolism, transcription, etc.

### Phylogenetic analysis

**Figure 2:**
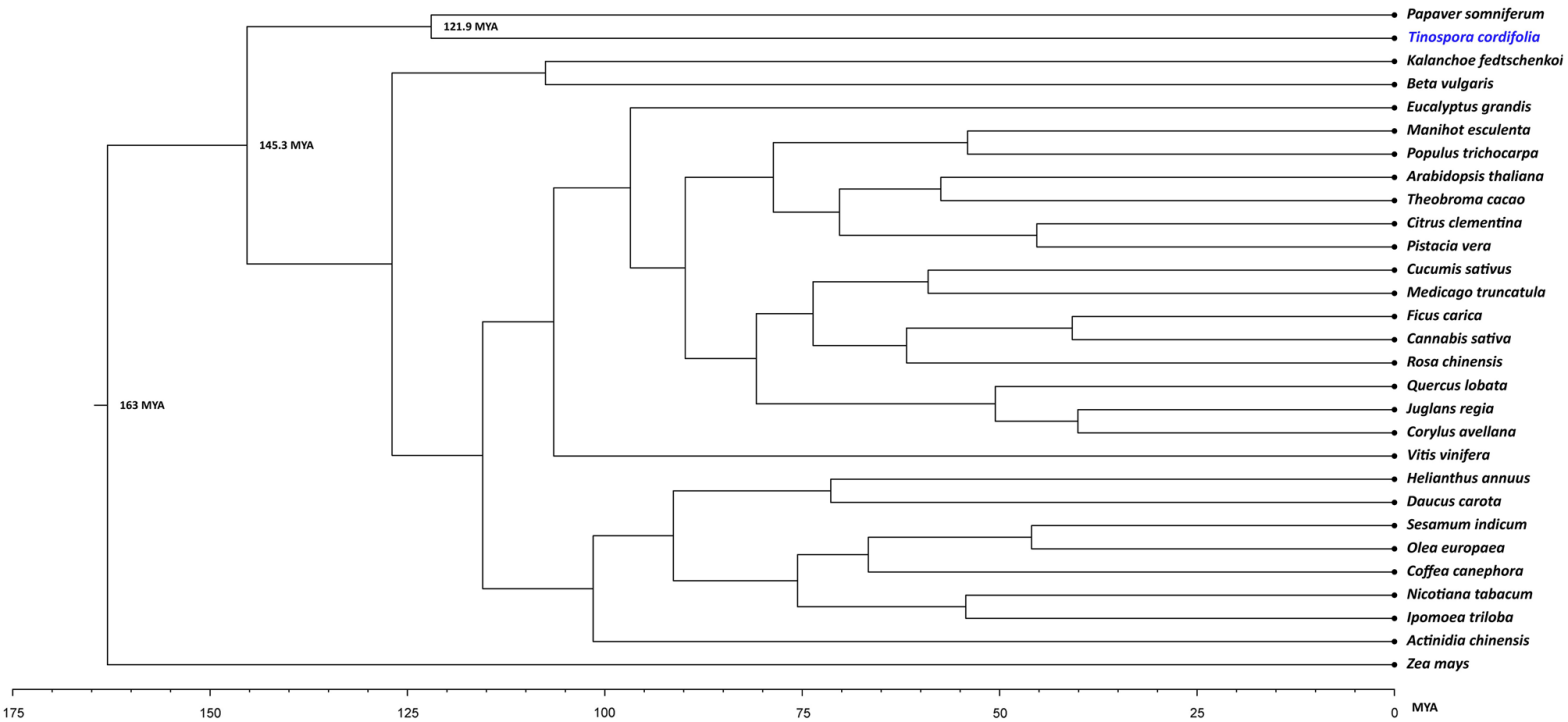
Phylogenetic tree showing the position of *T. cordifolia* with other species. The phylogenetic tree includes 26 core eudicot species, two basal eudicot species (*T. cordifolia* and *Papaver somniferum*) and a monocot species.

Among the identified 152,176 orthogroups, 224 fuzzy one-to-one orthogroups were predicted across 29 selected plant species. The filtered 224 fuzzy one-to-one orthogroups contained 201,201 alignment positions and were used to construct a phylogenetic tree. The position of *T. cordifolia* in the phylogenetic tree was found close to *Papaver somniferum* with estimated divergence time of ~ 122 MYA (95% CI), which also concurs with the species divergence time obtained from the TimeTree database^43^. *T. cordifolia* and *P. somniferum* form a separate clade from all the core eudicots and monocot species because the clade of *T. cordifolia* is basal eudicots that diverged very early from the core eudicots (145 MYA with 95% CI)^44,45^ (Fig. 2 and Supplementary Figure S1).

### Gene family contraction and expansion analysis

The CAFÉ analysis resulted in 5169 contracted and 782 expanded gene families in *T. cordifolia*. Among these gene families, 12 were highly contracted, and 25 were highly expanded in *T. cordifolia*. The highly contracted genes families were involved in phenylpropanoid biosynthesis, mitochondrial biogenesis, protein processing in endoplasmic reticulum, MAPK signalling pathway, and plant-pathogen interaction (Supplementary Table S9). The highly expanded gene families were majorly involved in phenylpropanoid biosynthesis, zeatin biosynthesis, carbohydrate metabolism, xenobiotic degradation, membrane trafficking, and MAPK signalling pathway (Supplementary Table S10).

### Orthologous gene clustering

**Figure 3:**
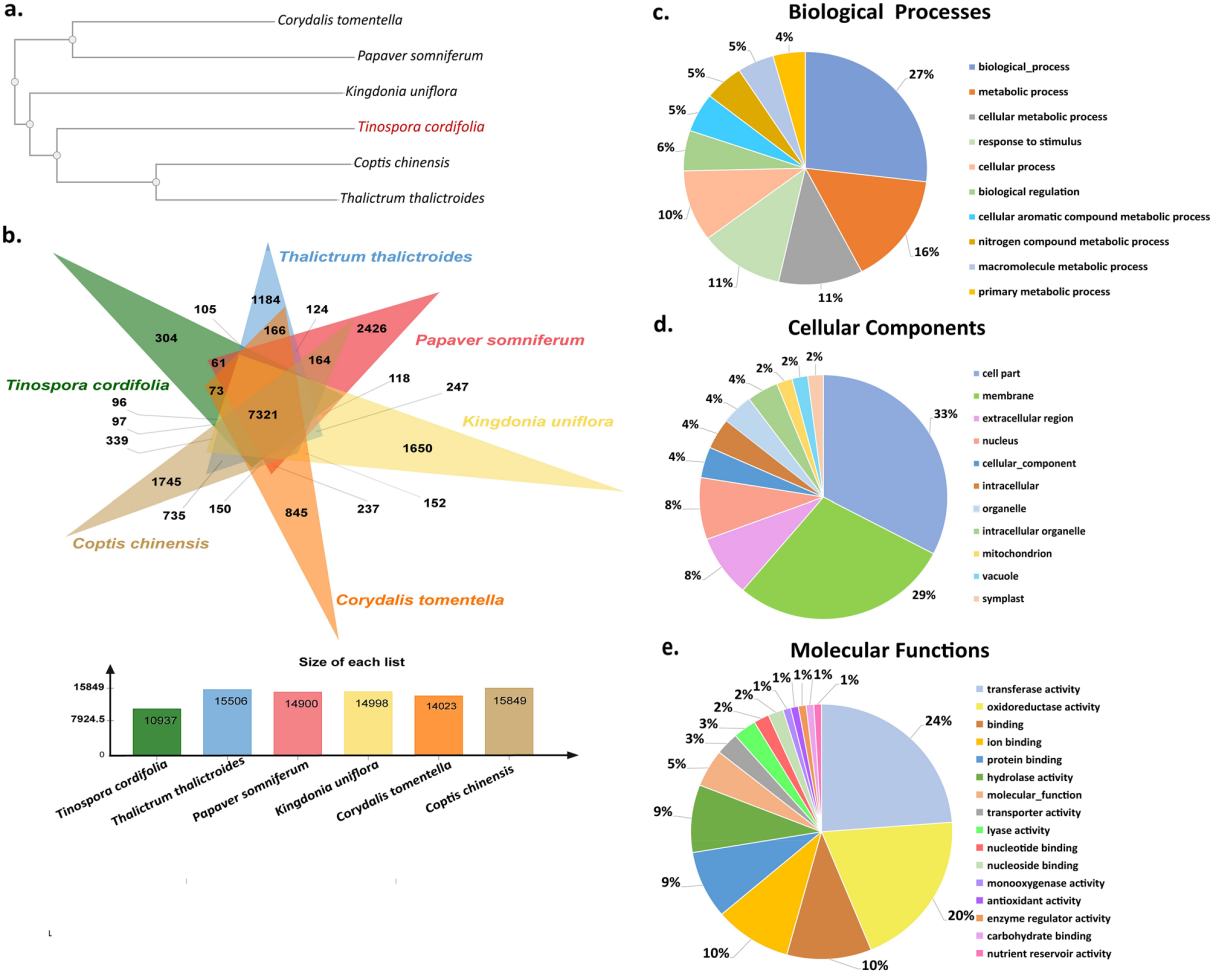
Comparative analysis of *T. cordifolia* genome. (**a**) Phylogeny of six Ranunculales species including *T. cordifolia* (**b**) Venn diagram of ortholog gene clustering of six species (**c**) Biological processes of *T. cordifolia* specific genes (**d**) Cellular component of *T. cordifolia* specific genes (**e**) Molecular functions of *T. cordifolia* specific genes Figs. 3a and 3b are obtained from Orthovenn3^138^.

The phylogenetic tree constructed using the peptide sequences of six Ranunculales species showed that *T. cordifolia* is closer to Ranunculaceae members i.e., *T. thalictroides* and *C. chinensis* compared to the other considered species (Fig. 3a). Among all the orthologous gene set, 66.9% (7321) gene set were shared across all six species, indicating their conservation. 2.7% (304) gene set were specific to *T. cordifolia* **(**Fig. 3b**)**. The GO functions of these species-specific genes are mentioned in Fig. 3c,d, and e.

### Genes with signatures of adaptive evolution

The comparative evolutionary analysis of the six selected species of order Ranunculales revealed 724 genes with high rate of evolution, 3432 genes with unique amino acid substitutions with functional impact and 3566 genes with positive selection in *T. cordifolia* (Supplementary Table S11 and Supplementary Table S12). Among these genes, 290 showed all three signatures of adaptive evolution in *T. cordifolia* (Supplementary Figure S2 and Supplementary Table S13).

### Benzyl iso-quinoline alkaloid (BIA) biosynthesis pathway in *T. cordifolia*

Alkaloids are the main bioactive compounds of Menispermaceae and Ranunculales plants. The genes involved in the BIA biosynthesis pathway were observed in *T. cordifolia* with transcriptomic evidences (Fig. 5, Supplementary Figure S3, and Supplementary Table S14). BIAs such as berberine and magnoflorine were also reported to have pharmaceutical activities against the COVID-19 virus^14,23^. Along with these, palmatine, tembetarine, and jatrorrhizine are the other pharmaceutically active biomolecules in *T. cordifolia*^7^. Among the genes involved in the biosynthesis of BIAs in *T. cordifolia*, two genes, Tyrosine decarboxylase (*TYDC*) and Canadine synthase (*CAS*), showed multiple signatures of adaptive evolution. Additionally, tyrosine aminotransferase (*TAT*) showed unique amino acid substitutions in *T. cordifolia*. The analysis of orthologous genes found seven genes (*4OMT*, *CNMT*, *CoOMT*, *RNMT*, *CST*, *NOMT*, and *SOMT*) with bacterial orthologs and three genes (*CNMT*, *RNMT*, and *TYDC*) with fungal orthologs (Supplementary Figures S4–18). *CAS* gene had orthologs from bryophytes and gymnosperms. *RNMT* had the highest number of bacterial and fungal orthologs among all the other BIA biosynthesis genes. Further, all genes except *CST* had orthologs from monocots.

### Other secondary metabolites biosynthesis pathway

Apart from BIA biosynthesis pathway, genes involved in terpenoid backbone biosynthesis, lignin biosynthesis, and flavonoid biosynthesis were found in *T. cordifolia* genome with transcriptomic evidences (Supplementary Figure S3). Among the 19 genes involved in terpenoid backbone biosynthesis, eight genes showed at least one signature of adaptive evolution. Out of the eight genes, *DXS, AACT* and *FPPS* showed MSA, *PMK,* and *FOLK* showed positive selection, *ispE* and *GGPS1* showed unique amino acid substitutions, and *ispH* showed high nucleotide divergence (Fig. 5, Supplementary Table S15, and Supplementary Figure S3).

Genes *PAL, C4H*, and *4CL*, involved in both flavonoid and lignin biosynthesis pathways, showed signatures of adaptive evolution in *T. cordifolia*. Along with these three genes, five other genes of flavonoid biosynthesis showed at least one signature of adaptive evolution. Among these eight genes, *PAL*, *4CL*, *DFR*, and *ANR* showed multiple signatures of adaptive evolution. *UFGT* showed only positive selection, and *C4H*, *F3H* and *FLS* showed only unique amino acid substitutions. Among the 13 genes involved in lignin biosynthesis, eight genes showed at least one signature of adaptive evolution. *PAL*, *4CL*, *C3H*, *CCR*, and *CAD* showed multiple signatures of adaptive evolution. *HCT* and *CCoAOMT* showed only positive selection, and *C4H* showed only unique amino acid substitutions (Supplementary Table S15 and Supplementary Figure S3).

### Common symbiosis signaling pathway (CSSP) in *T. cordifolia*

Several plant parts of *T. cordifolia* have been associated with endosymbiotic microorganisms^35,37,46^. Therefore, we traced the genes involved in CSSP and found signatures of adaptive evolution in these genes. The angiosperm-specific conserved genes (*Castor*, *EPP1*, *kinF*, *LIN*, *SYN*, and *VAPYRIN*) responsible for plant endosymbiosis were present in *T. cordifolia*. However, all the land plant-specific conserved genes (*CCaMK*, *RAD*, *STR*, *STR2*, and *SymRK*) for plant endosymbiosis except *CYCLOPS* were also found in *T. cordifolia*^47^. Among these conserved genes, *SYN* showed positive selection in *T. cordifolia*. Twenty-four genes showed signatures of adaptive evolution, of which *NUP133*, *NENA*, *MCA8*, *D3*, *DIS*, *MSBP1*, *Skl1*, *SUNN*, *RFCb*, and *CCD7* showed multiple signatures of adaptive evolution. Among these 24 genes, *LYK3*, *CNCG15, SYP132A*, *MIG1*, *KIN3* and *SUT2* showed only positive selection, and *NSP1*, *D14, FatM, LOM1, VAMP721d*, *HA1*, *EXO70I*, and *VTI12* showed only unique amino acid substitutions (Fig. 6 and Supplementary Table S16).

### Adventitious root associated genes in *T. cordifolia*

Adventitious roots are aerial roots that emerge from the plant shoot system and function as storage, support, etc.^48^. *T. cordifolia*, being a climbing plant, bears assimilatory roots (a type of adventitious roots) that help plants anchorage on other trees or structures, and perform photosynthesis^49,50^. 25 genes associated with adventitious root development in plants showed signatures of adaptive evolution in *T. cordifolia* (Supplementary Table S17). Among these 25 genes, a few genes also showed evidence of their gene expression in *T. cordifolia* (Supplementary Figure S3). Out of the 25 genes, 17 genes showed MSA, and eight showed only one signature of adaptive evolution. Among these genes, 10 were involved in auxin biosynthesis, transport, and response. Thirteen genes that showed signatures of adaptive evolution were involved in the formation of meristem and primordium. Genes *PRP1* and *CEP1*, involved in cell wall modification, showed two and three signatures of adaptive evolution, respectively. Additionally, an auxin-responsive gene *SAUR15* was highly expanded in *T. cordifolia* (Supplementary Table S17). Additionally, three miRNAs associated with adventitious root formation were present in *T. cordifolia*.

### Peroxisome associated genes in *T. cordifolia*

Peroxisomes play key roles in several processes crucial to plant growth and development. It plays an essential role in fatty acid β-oxidation, phytohormones biosynthesis (auxin, salicylic acid and jasmonic acid), photorespiration, transportation of metabolites and cofactors, etc.^51^. Fourteen peroxisome-associated genes were found with at least one signature of adaptive evolution and a few genes among them also showed gene expression in *T. cordifolia* (Supplementary Table S18 and Supplementary Figure S3). All 14 genes showed unique amino acid substitutions except solute carrier family 25 member 17 (*PMP34*) and *PEX-19*, which showed positive selection. Among these 14 genes, *ALD* (member 3) and catalase showed only unique amino acid substitutions in *T. cordifolia*. Except these three genes, all other genes (11) showed MSA. Among these 11 MSA genes, five genes (*PEX5*, *PEX7*, *PEX14*, acyl-CoA oxidase, and L-pipecolate oxidase) showed all three signatures of adaptive evolution. Four genes, long-chain acyl-CoA synthetase, *PEX-3*, and two superoxide dismutases, showed only two signatures of adaptive evolution. Among these 14 genes, *PEX3, PEX5*, *PEX7, PEX14, PEX19, MPV17,* and *PMP34* were involved in peroxisome biogenesis (Fig. 7 and Supplementary Table S18).

### Plant growth, phytohormones and stress tolerance associated genes in *T. cordifolia*

76 genes with all three signatures of adaptive evolution were associated with plant growth and development involved in leaf development, root development, fruit ripening, tissue development, pollen germination, flowering regulation, seed development, cell wall modification, etc. Among these, ten genes were associated with root development in *T. cordifolia*. 28 genes that were associated with phytohormones such as auxin, gibberellin, abscisic acid, ethylene, jasmonic acid, salicylic acid, cytokinin, and brassinosteroids showed all three signatures of adaptative evolution. Among these, nine genes were associated with auxin, and eight genes were associated with abscisic acid. Among plant stress tolerance-associated genes, 49 and 34 genes were involved in abiotic and biotic stress tolerance, respectively. These 49 abiotic stress tolerance genes were involved in light/heat tolerance, cold tolerance, salt tolerance, osmotic stress tolerance and oxidative stress regulation and tolerance (Supplementary Table S19).

## Discussion

*Tinospora cordifolia* is known to produce several phytochemicals as secondary metabolites that are responsible for its various medicinal properties^11,52^. Being a medicinally important herb with therapeutic applications in multiple health conditions, the genome and transcriptome sequencing of *T. cordifolia* was much needed to gain genomic insights into the secondary metabolites production responsible for its medicinal properties. This study reports the draft genome assembly of *T. cordifolia* which is also the genome from the *Tinospora* genus and its family. This study also deliberates the genome assembly, transcriptome assembly, gene set, and phylogeny of *T. cordifolia* that will help in understanding the medicinal properties of this multipurpose herbal plant. The genome assembly was constructed using a hybrid approach that has emerged as a promising methodology to significantly increase the contiguity in the genome assembly by increasing scaffold N50 and decreasing the number of scaffolds compared to short-read technology. This hybrid approach helped in the sequencing and assembly of this complex genome of *T. cordifolia* with high heterozygosity and high repetitive content^53^. Further, this observed high heterozygosity of *T. cordifolia* could also be associated with its dioecious nature as reported in earlier studies^54–56^.

*T. cordifolia* belongs to the order Ranunculales and our phylogenetic tree finds its position as a distinct branch separate from all the considered core eudicots and the monocot (Fig. 2). This could be due to the early divergence of order Ranunculales from all the other core eudicots. Order Ranunculales is regarded as an early diverging eudicot order and is among a few other eudicot orders (collectively known as basal eudicots) that are found to be sister lineage to the core eudicots, which was also observed in the case of *T. cordifolia* that showed early divergence from all other dicot species^44,57,58^. According to the phylogenetic tree, *T. cordifolia* was found in a separate clade as basal eudicot with *P. somniferum* (122 MYA) diverged from core eudicots (145 MYA), which was also supported by other studies (Fig. 2)^59–64^*.*

**Figure 4:**
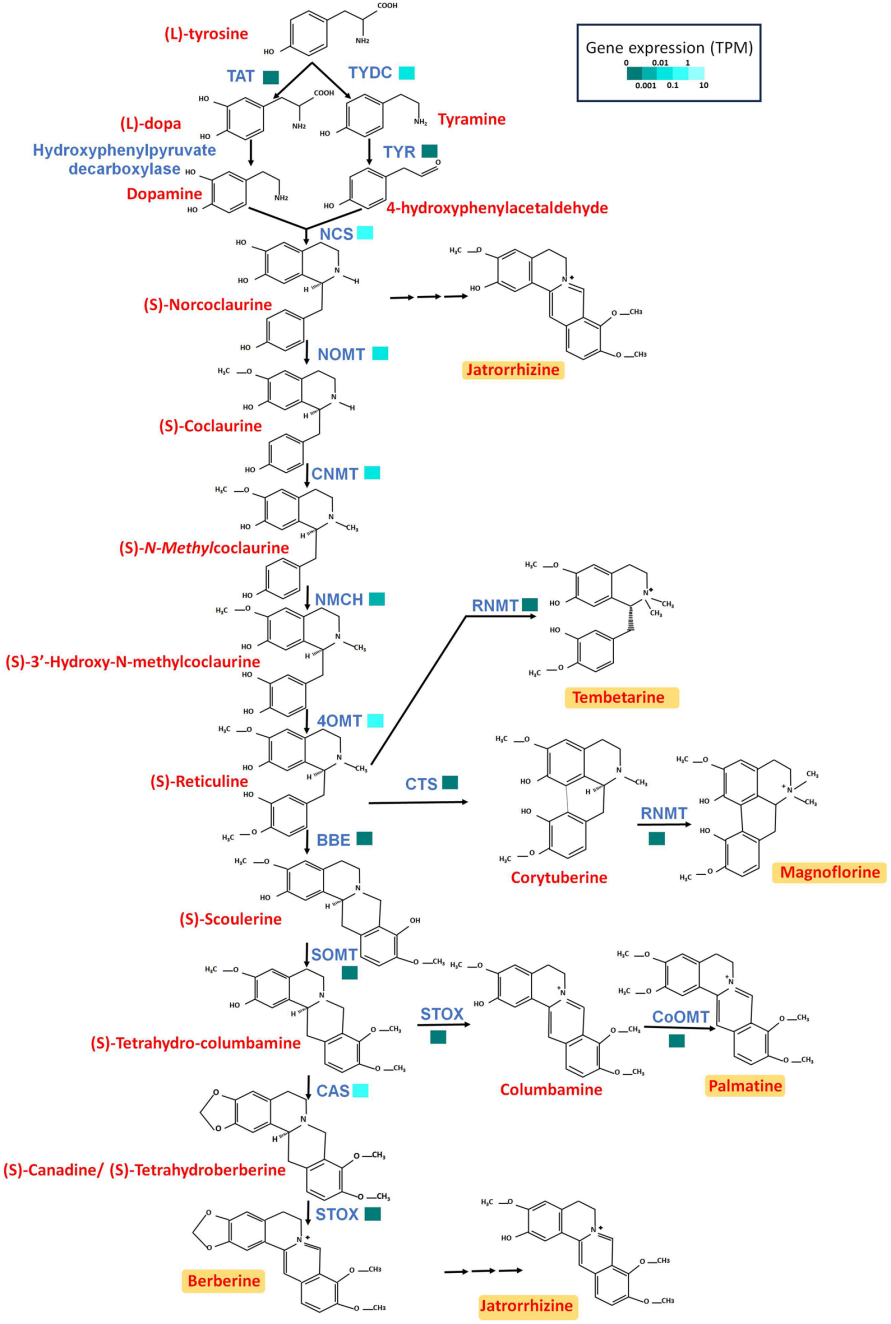
Benzyl iso-quinoline alkaloid biosynthesis pathway in *T. cordifolia.* This pathway indicates the genes involved in biosynthesis of alkaloids like berberine, magnoflorine, palmatine, and tembetarine. It includes tyrosine aminotransferase (*TAT*), Hydroxyphenylpyruvate decarboxylase, Tyrosine decarboxylase (*TYDC*), Tyrosinase/Phenolase (*TYR*/*3OHase*), (S)-Norcoclaurine synthase (*NCS*), Norcoclaurine 6-O-methyltransferase (*NoMT*), (S)-Coclaurine N-methyltransferase (*CNMT*), N-Methylcoclaurine 3'-hydroxylase (*NMCH*), 3'-Hydroxy-N-methylcoclaurine 4' -O-methyltransferase (*4OMT*), Corytuberine synthase (*CTS*), (S)-Corytuberine-N-methyltransferase (*SCNMT*/*RNMT*), Berberine bridge enzyme (*BBE*), S)-Scoulerine 9-O-methyltransferase (*SOMT*/*SMT*), (S)-Tetrahydroprotoberberine oxidase (*STOX*), Columbamine O-methyltransferase (*CoOMT*), and (S)-Canadine synthase (*CAS*).

**Figure 5:**
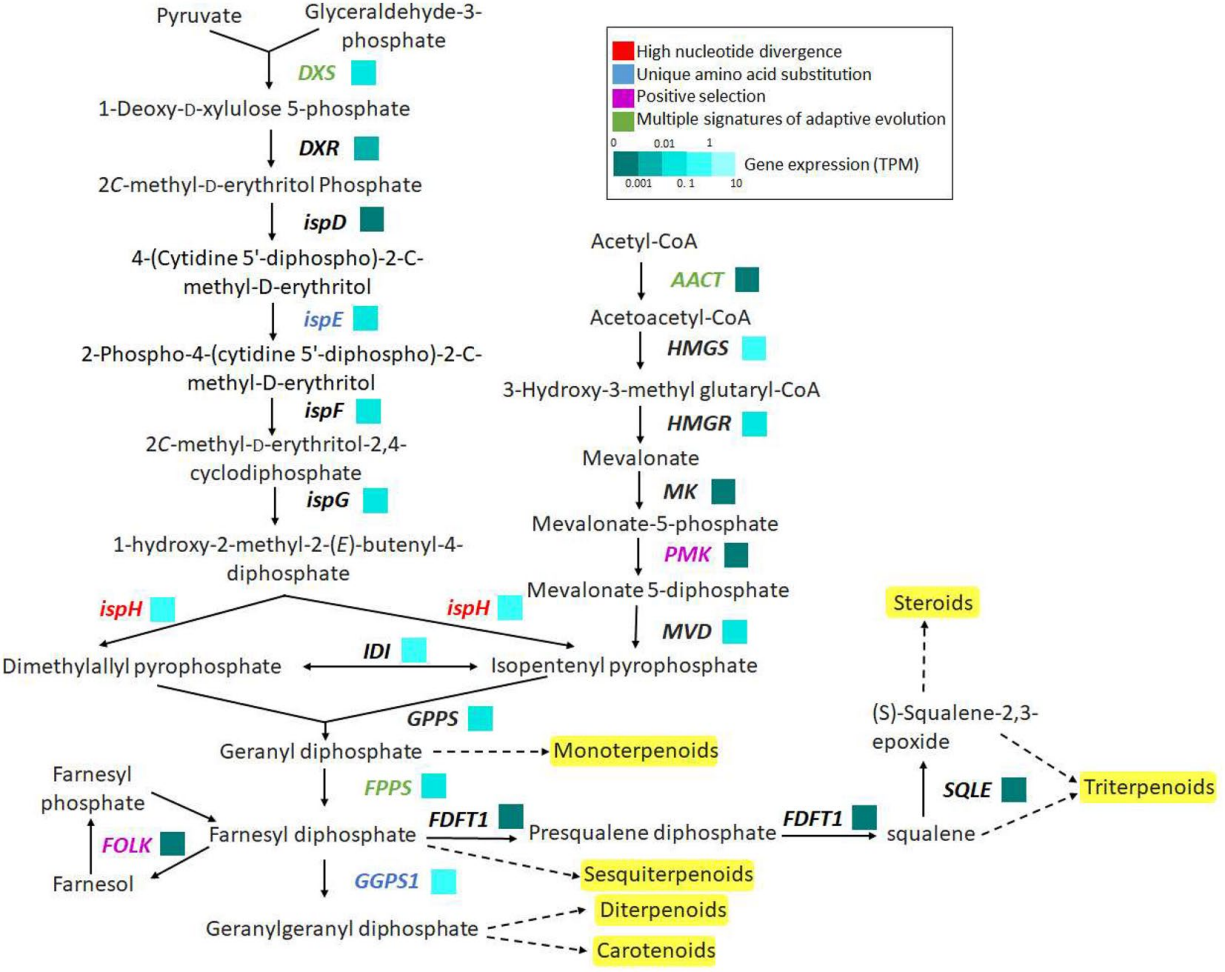
Terpenoid biosynthesis pathway in *T. cordifolia.* This pathway indicates the genes involved in biosynthesis of terpenoids in *T. cordifolia*. It includes 1-deoxy-D-xylulose-5-phosphate synthase (*DXS*), 1-deoxy-D-xylulose-5-phosphate reductoisomerase (*DXR*), 2-C-methyl-D-erythritol 4-phosphate cytidylyltransferase (*ispD*), 4-diphosphocytidyl-2-C-methyl-D-erythritol kinase (*ispE*), 2-C-methyl-D-erythritol 2,4-cyclodiphosphate synthase (*ispF*), (E)-4-hydroxy-3-methylbut-2-enyl-diphosphate synthase (*ispG*), 4-hydroxy 3-methylbut-2-en-1-yl diphosphate reductase (*ispH*), Acetoacetyl-CoA thiolase (*AACT*), HMG-CoA synthase (*HMGS*), HMG-CoA reductase (*HMGR*), Mevalonate kinase (*MK*), Phosphomevalonate kinase (*PMK*), Mevalonate-5-diphosphate decarboxylase (*MDD*), Isopentenyl diphosphate isomerase (*IDI*), Geranyl diphosphate synthase (*GPPS*), Farnesyl diphosphate synthase (*FPPS*), Geranylgeranyl diphosphate synthase (*GGPS*), Farnesol kinase (*FOLK*), Farnesyl-diphosphate farnesyltransferase 1 (*FDFT1*), and Squalene monooxygenase (*SQLE*). Genes in red, blue, pink and green represents genes showing high nucleotide divergence, unique amino acid substitutions, positive selection, and multiple signatures of adaptive evolution, respectively.

Alkaloids, terpenoids, lignans, and flavonoids are the main secondary metabolites in *T. cordifolia.* Among the alkaloids, benzyl iso-quinoline alkaloid compounds like berberine, palmatine, magnoflorine, and tembetarine are the key bioactive compounds in *T. cordifolia*. Among the BIAs, berberine and magnoflorine have been reported to have immunomodulatory, antibacterial, and anti-viral activities^14,16,65^. Among a few tested compounds from *T. cordifolia*, berberine was reported to have the highest affinity against SARS-CoV-2 targets^66^. It also inhibited DNA and protein synthesis in bacteria^65^. Another BIA, tembetarine, possesses anti-diabetic and antibacterial activities, whereas columbamine has immunomodulatory activity^16,67^. Additionally, its terpenoids and flavonoids are reported to possess antidiabetic activity, whereas lignans are reported to have anti-oxidant activity in *T. cordifolia*^8,11^. These alkaloids, terpenoids, lignans, flavonoids along with various other phytochemicals, thus contribute to the diverse medicinal properties of *T. cordifolia*. Therefore, we traced the genes involved in the biosynthesis pathways of benzyl iso-quinoline alkaloids, terpenoids, lignans, and flavonoids in *T. cordifolia*, and found signatures of adaptive evolution along with transcriptomic evidences in a few of these genes (Figs. 4, 5 and Supplementary Figure S3). The genes with signatures of adaptive evolution involved in BIA (*TAT*, *TYDC* and *CAS*) and terpenoids (*DXS, AACT* and *FPPS*) are also reported in other plant species^64,68–71^. The lignin and flavonoid biosynthesis genes with evolutionary signatures (*PAL*, *4CL*, *C3H*, *CCR*, and *CAD)* are also reported to have significance in other plant genomes^72–75^. The observed evolutionary signatures in genes involved in these pathways might contribute to the production of diverse secondary metabolites in *T. cordifolia*.

**Figure 6:**
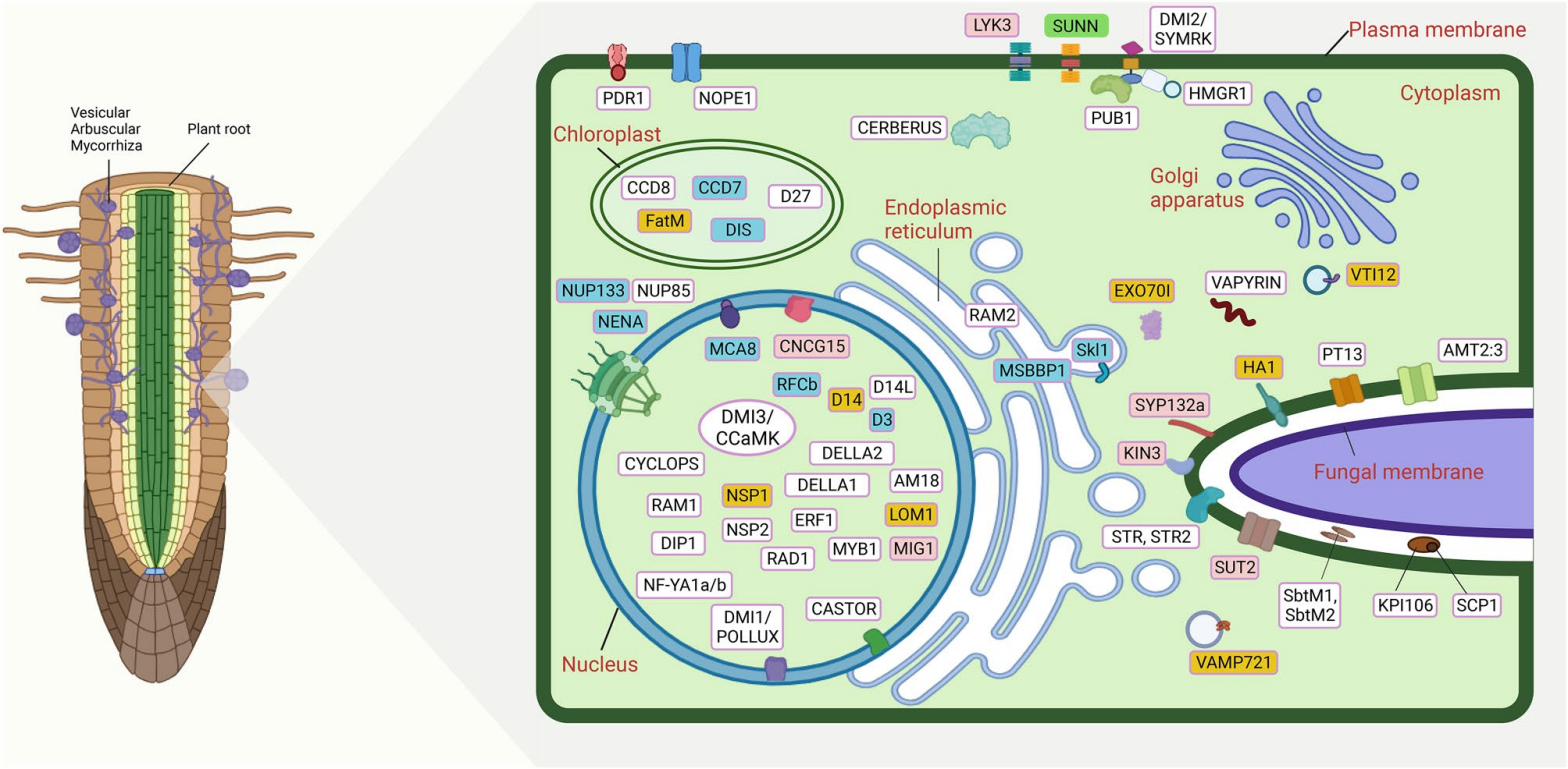
Common symbiosis signalling pathway in *T. cordifolia.* The figure (Created with BioRender.com) indicates the key genes of common symbiosis signalling pathway and the fill name of genes is provided in the Supplementary Table S16. Genes in pink indicates genes only with positive selection, yellow indicates genes showing only amino acid substitutions, blue indicates genes with two signatures, and green indicates genes with all three signatures of adaptive evolution.

The presence of endophytic fungi in plants helps in the availability of essential mineral nutrients, which improve plant growth via phytohormone production, tolerance against stressed conditions like drought, salinity, heavy metals, high or low temperatures, etc.^33,76,77^. However, among the 12 conserved Arbuscular Mycorrhiza (AM) endosymbiosis-associated genes in plants, only one gene *CYCLOPS* is found to be absent in *T. cordifolia,* which was similar to other AM endosymbiosis-associated plant species such as *Fragaria x ananassa*, *Castanea mollissima* and *Quercus robur*^47^. Most genes mentioned above are directly involved in CSSP, which is responsible for AM endosymbiosis in plants. The genes involved in this pathway showed signatures of adaptive evolution in *T. cordifolia,* which indicates towards the evolution of AM symbiosis (Fig. 6 and Supplementary Table S16). These genes with evolutionary signatures were also reported in other plant species and associated with endosymbiosis^78,79^. The AM endosymbiosis is also influenced by phytohormones like auxin, cytokinin, jasmonic acid, brassinosteroids, etc.^80,81^. Thus, the genes associated with these phytohormones also showed signatures of adaptive evolution which further suggests the evolution of AM symbiosis in *T. cordifolia* (Supplementary Table S13). Moreover, endophytic bacteria and fungi present in *T. cordifolia* are *Bacillus* spp., *Aneurinibacillus* spp., *Pseudomonas* spp., *Penicillium* spp., *Nigrospora oryzae*, *Chaetomium globosum*, *Colletotrichum* spp., *Curvularia* spp., *Alternaria alternata*, *Phome* spp., etc.^36,37^. These endophytes are known to produce secondary metabolites, and phytohormones like indole acetic acid and gibberellins that helps in the growth and stress tolerance of their host plants^82–91^. Thus, evolutionary signatures in genes associated with common symbiosis signalling pathway might contribute to the evolution of medicinal properties in *T. cordifolia*.

**Figure 7:**
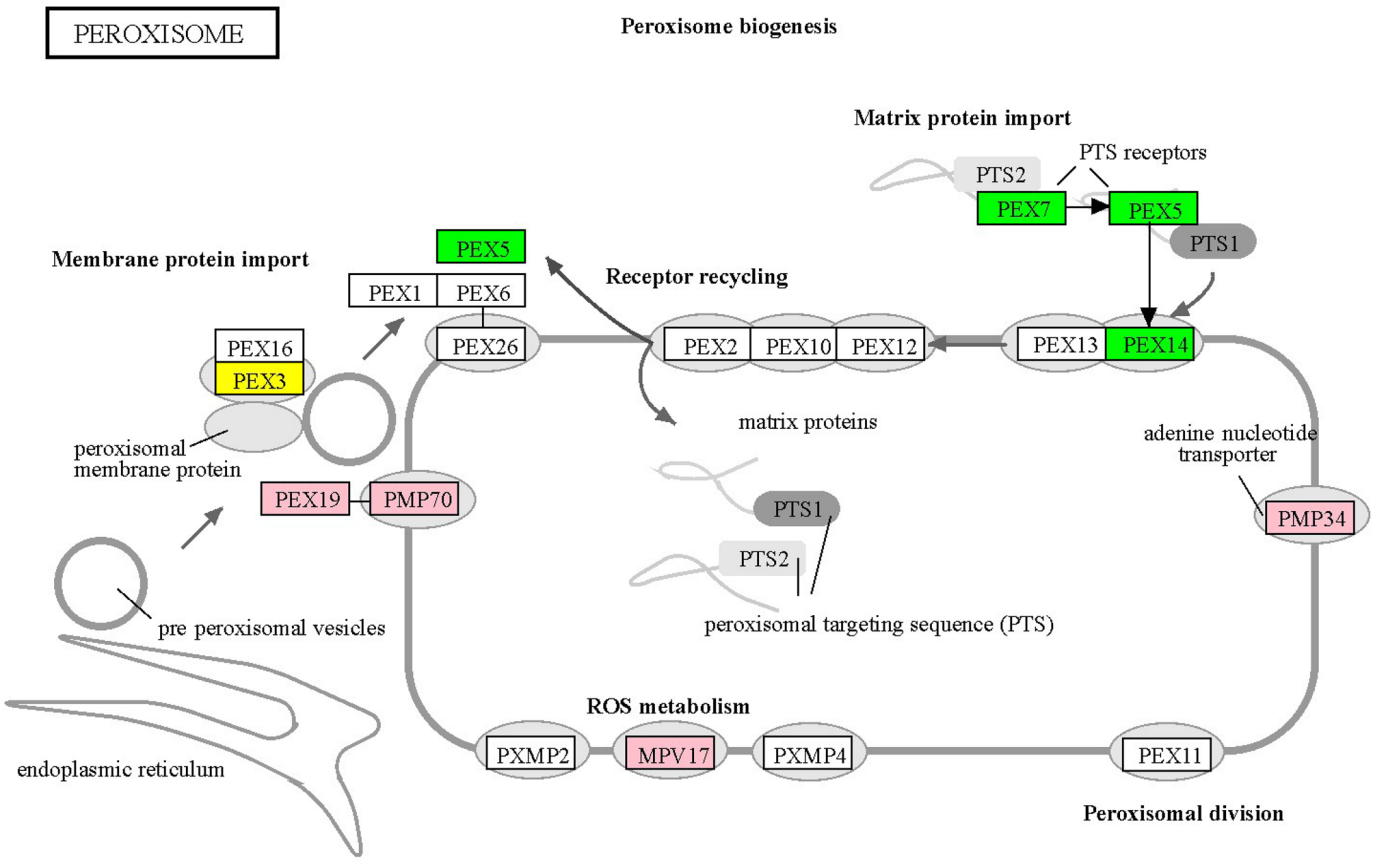
Peroxisome biogenesis pathway in *T. cordifolia.* The figure of peroxisome biogenesis is generated from KEGG database^137^. It includes genes *PEX1, PEX2, PEX3, PEX5, PEX6, PEX7, PEX10, PEX11, PEX12, PEX13, PEX14, PEX16, PEX19, PEX26, PXMP2, MPV17, PXMP4*, and *PMP34*. Genes in pink, yellow and green colour indicate genes showing one, two and all three signatures of adaptive evolution, respectively.

*T. cordifolia,* being a climbing plant, remains in the shades of the supportive tree where lesser sunlight might reach the plant^49^. Thus, these plants increase their photosynthesis capacity and anchorage by forming assimilatory roots. These aerial roots perform photosynthesis and provide anchorage that contributes to plant growth^48^. The adventitious roots in *T. cordifolia* were found to be evolved due to the presence of signatures of adaptive evolution in these genes involved in adventitious root formation (Supplementary Table S17). These genes with evolutionary signatures were also reported in other plant species and play key roles in adventitious root formation^92–94^. Additionally, phytohormones also play an important role in regulating adventitious root formation in plants^95^. Along with the genes involved in adventitious root formation, phytohormone associated genes were also found to be evolved in *T. cordifolia*. Peroxisomes play an important role in photorespiration and plant growth that occurs in all C3 plants^51^. *T. cordifolia*, a C3 plant, performs photorespiration and lacks adaptations like the CAM pathway of CAM plants. Therefore, the signatures of adaptive evolution observed in genes associated with peroxisome biogenesis appear to be significant for C3 photosynthesis contributing to plant growth in *T. cordifolia* (Fig. 7 and Supplementary Table S18). These evolutionary signatures in genes associated with adventitious roots, phytohormones, and peroxisomes contribute towards the overall growth of *T. cordifolia*. Taken together, the comprehensive comparative evolutionary analysis helped to reveal the evolution in genes involved in common symbiosis signalling pathway, adventitious root formation, peroxisome associated, abiotic and biotic stress tolerance, and in genes associated with various phytohormones; thus, providing genomic clues on the diverse medicinal properties of this species.

The study reports the draft genome of *T. cordifolia* and provides important insights on the genomic basis of its medicinal properties due to the presence of diverse secondary metabolites. However, the presence of these metabolites also posed problems during the DNA and RNA extraction steps, and also severely reduced the data throughput from the ONT nanopore sequencing technologies. Thus, it is required to develop improved nucleic acid extraction protocols to deal with such medicinal plants. Further, the presence of high heterozygosity and repetitive content in *T. cordifolia* genome posed challenges during the assembly process and limited the assembly statistics such as the N50 score. The availability of more data from both second and third generation sequencing technologies is much needed to achieve a better coverage and a more contiguous assembly of this species. Further, due to the absence of genomes from the *Tinospora* genus and Menispermaceae family, species belonging to the same order were used for evolutionary analysis to eliminate the effect of large genetic distances. The availability of more genomes from closely related plant species would offer a deeper understanding of the species-specific or family-specific characteristics of *T. cordifolia*. Additionally, conducting experimental validation on the role of adaptively evolved genes in secondary metabolites biosynthesis will provide further support to the results of these analysis and will help in developing better approaches to benefit from the medicinal properties of this plant.

## Conclusion

The insights obtained from this study and the availability of the genome sequence of *T. cordifolia* will help bridge the missing link between its genomic and medicinal properties and provide leads for exploring the genomic basis of these properties. It will also aid in various comparative genomic studies and act as a reference for future species sequenced from its genus and family. It will also help in the genome-wide phylogenetic assessments and evolutionary analyses of this species. The knowledge of mechanisms and pathways involved in producing its numerous medicinally important secondary metabolites will help better exploit these pathways and resultant metabolites for medicinal purposes and therapeutic applications.

## Methods

### Sample collection, species identification, nucleic acids extraction, and sequencing

The plant was brought from a nursery in Bhopal, Madhya Pradesh, India (23.2599° N, 77.4126° E). All methods were performed in accordance with the national guidelines concerning research involving plants. The leaves from the young plant were cleaned and used for DNA and RNA extractions. For DNA extraction, the leaves were homogenized in liquid nitrogen. For genomic DNA extraction, the powdered leaf was washed with 70% ethanol and distilled water to eliminate any such compounds that may hinder the extraction process, and employed CTAB-based lysis buffer for the isolation^96^. Two genes: one nuclear gene and one chloroplast gene (internal transcribed spacer and Maturase K, respectively), were used for the species identification. These genes were amplified and sequenced at an in-house Sanger sequencing facility. The total RNA was extracted with TRIzol reagent from the leaf sample using multiple reactions and the RNA was then pooled before proceeding for the library preparation^97^. The TruSeq Stranded Total RNA library preparation kit with Ribo-Zero Plant workflow (Illumina, Inc., USA) was deployed for preparing the transcriptomic library. The genomic library for linked reads was prepared using a Gel Bead kit and Chromium Genome library kit on a Chromium Controller instrument (10× Genomics, CA, USA). The quality of both the libraries (transcriptomic and genomic) was checked on Tapestation 4150 (Agilent, Santa Clara, CA) and sequenced on an Illumina platform, NovaSeq 6000 (Illumina, Inc., USA) for producing paired-end reads. For Nanopore sequencing, the genomic DNA libraries were prepared using SQK-LSK109 and 110, and sequenced on MinION sequencer. The comprehensive DNA and RNA extraction procedure is mentioned in the Supplementary Text.

### Genome assembly

An array of Python scripts (https://github.com/ucdavis-bioinformatics/proc10xG) were used to remove the barcode sequences from the 10× Genomics linked reads. GenomeScope v2 was employed to calculate heterozygosity similar to other studies and SGA-preqc for genome size estimation of *T. cordifolia* using the k-mer count distribution method^98–100^. To reduce the impact of sequencing errors on the estimation, only the k-mers with higher occurrences are considered in this k-mer count distribution method. Initially, the sga preprocess was run with the option "–pe-mode 1" to consider all the paired-end barcode-filtered 10× Genomics reads. Following this step, the pre-processed reads were indexed by sga index with ‘ropebwt’ algorithm and "–no-reverse" options. Finally, the SGA-preqc was run with default options for the genome size estimation. The de novo assembly was generated by Supernova assembler v.2.1.1 (with maxreads = all options and other default settings) using 499.36 million raw reads^101^. The ‘pseudohap2’ style in Supernova mkoutput was implemented to assemble the haplotype-phased genome.

The barcodes of linked reads were processed using Longranger basic v2.2.2 (https://support.10xgenomics.com/genome-exome/software/pipelines/latest/installation) and these processed reads were used by Tigmint v1.2.1 to rectify the misassemblies present in Supernova assembled genome^102^. AGOUTI v0.3.3 with quality-filtered transcriptome reads was used to accomplish the initial scaffolding^103^. To construct a more contiguous assembly, ARCS v1.1.1 with the barcode-processed linked reads was used to provide additional scaffolding and enhance the contiguity of the genome assembly^104^. LINKS v1.8.6 was also used with the adapter-filtered Nanopore reads (using Porechop) for additional scaffolding^105^. LR_Gapcloser used Nanopore reads to perform gap-closing of the assembly^106^. Using a bloom filter-based method and k-mer value ranging from 30 to 120 with a ten bp interval, Sealer v2.1.5 used the linked reads (barcode processed) for gap-closing in the assembly^107^. Performing scaffolding multiple times could give rise to local misassemblies, small indels, or distinct base errors that were overwhelmed using Pilon v1.23 which utilized the linked reads (barcode-processed) to increase the assembly quality^108^. The obtained assembly was length based filtered such that scaffolds with length ≥ 2000 bp were retained. The completeness of genome assembly was evaluated with BUSCO v5.4.4 which used viridiplantae_odb10 database for the assessment^109^.

### Transcriptome assembly

The de novo transcriptome assembly was carried out using RNA-Seq data generated in this study. Trimmomatic v.0.38 was used to process raw data reads, i.e., adapter removal and quality filtration^110^. The de novo transcriptome assembly was constructed using Trinity v2.9.1 with a strand-specific option and other default parameters using the processed paired-end reads^111^. A Perl script offered in Trinity software package was utilized to evaluate the assembly statistics.

### Genome annotation

The genome annotation was performed on the polished assembly. RepeatModeler v2.0.1 used this genome to construct a de novo repeat library^112^. The obtained repeat sequences were clustered using CD-HIT-EST v4.8.1 with sequence identity as 90% and 8 bp seed size^113^. Repeat library obtained from RepeatModeler was further curated using TEclass2 software with a probability threshold of > 0.65^114^. Using this curated repeat library, RepeatMasker v4.1.0 (RepeatMasker Open-4.0, http://www.repeatmasker.org) soft-masked the genome that was used for the construction of the gene set. MAKER pipeline that employs ab initio*-*based gene prediction programs and evidence-based approaches to predict the final gene model was used for genome annotation^115^. As empirical evidence in the MAKER pipeline, the de novo transcriptome assembly of *T. cordifolia* and protein sequences of its phylogenetically closer species were used. The ab initio gene prediction, evidence-based alignments, and polishing of alignments were achieved using AUGUSTUS v3.2.3, BLAST and Exonerate v2.2.0, respectively, with the MAKER pipeline^116,117^. The coding genes were filtered based on the criteria of AED (Annotation Edit Distance) value < 0.5 and gene length ≥ 150 bp^118^. This coding gene set was annotated against NCBI-nr and Swiss-Prot database using blastp (e-value 10^–5^), and against Pfam-A database using HMMER v3.1 (e-value 10^–5^)^116,119–121^. The completeness of genome annotation was evaluated with BUSCO v5.4.3 which used viridiplantae_odb10 database for the assessment^109^. de novo tRNAs prediction, de novo rRNAs prediction, and miRNAs identification (homology-based) were performed using tRNAscan-SE v2.0.7, Barrnap v0.9 (https://github.com/tseemann/barrnap) and miRBase database, respectively^122–124^.

### Functional annotation

*T. cordifolia* high-confidence gene set was annotated using blastp (e-value 10^–5^) with NCBI non-redundant (nr) and SWISS-PROT databases, and HMMER v3.3 with Pfam-A v32.0 database^116,119,120,125^. This coding gene set was functionally annotated and assign Gene Ontology (GO) categories, Cluster of Orthologous Groups (COG) categories, and KEGG pathways using WebGestalt web server, eggNOG-mapper v2.1.12, and KAAS v2.1, respectively^126–128^.

### Phylogenetic analysis

Among all the eudicot species accessible on Ensembl Plants release 56, a total of 27 species were selected by choosing one species from each offered order. The 27 eudicot species and an outgroup species, *Zea mays*, were used to identify orthologs^129^. The 28 selected species, along with the outgroup species, were—*Actinidia chinensis*, *Arabidopsis thaliana*, *Beta vulgaris, Cannabis sativa, Citrus clementina, Coffea canephora, Corylus avellana, Cucumis sativus, Daucus carota, Eucalyptus grandis, Ficus carica, Helianthus annuus, Ipomoea triloba, Juglans regia, Kalanchoe fedtschenkoi, Manihot esculenta, Medicago truncatula, Nicotiana attenuata, Olea europaea*, *Papaver somniferum*, *Pistacia vera, Populus trichocarpa, Quercus lobata, Rosa chinensis, Sesamum indicum, Theobroma cacao*, *Vitis vinifera,* and *Zea mays.*

The orthogroups were formed using the proteome files of 27 selected eudicot species along with *Zea mays* and MAKER retrieved protein sequences of *T. cordifolia*. Among all the protein sequences, the longest isoforms were retrieved for all the species and provided to OrthoFinder v2.5.4 for orthogroups construction^130^. The orthogroups comprising genes from all 29 species were retrieved from all the identified orthogroups. KinFin v1.1 was used to increase the genes in one-to-one orthogroups that identified and extracted fuzzy one-to-one orthogroups among these retrieved orthogroups^131^. In cases where multiple genes were present for a single species in any orthogroup, the longest gene among them was selected as representative.

MAFFT v7.310 was used to discretely align all the identified fuzzy one-to-one orthogroups to construct the phylogenetic tree^132^. The multiple sequence alignments were trimmed to eradicate empty sites, and the alignments were concatenated using BeforePhylo v0.9.0 (https://github.com/qiyunzhu/BeforePhylo). The concatenated alignments were used by RAxML v8.2.12, based on a rapid hill climbing algorithm, to create the maximum likelihood-based phylogenetic tree (100 bootstrap values and amino acid substitution model ‘PROTGAMMAAUTO’)^133^. Similarly, another phylogenetic tree was constructed using a basal angiosperm species i.e., *Amborella trichopoda* as an outgroup instead of *Zea mays*. Additionally, MCMCtree was implemented to estimate the time of divergence between species with -nsample 1,000,000 and -burnin 10,000, and two calibration point was taken between basal eudicots—eudicots divergence (126–132 MYA) and between monocot—eudicot (142–163 MYA) from TimeTree database v5^43,134^.

### Gene family expansion and contraction analysis

The proteome files from 29 selected species and the above-generated phylogenetic tree were used to analyse the evolution of gene families using CAFÉ v5^135^. The proteome files provided contained the longest isoform of each protein for all 29 species. Using TimeTree database v5.0, the divergence time between *T. cordifolia* and *Beta vulgaris* was obtained. This obtained time was used as a calibration point for adjusting the phylogenetic tree to an ultrametric species tree^43^. These protein sequences were aligned using blastp in all-versus-all mode^116^. The clustering of blastp results was performed using MCL v12-137^136^. Gene families with genes from < 2 species of the specified clades and ≥ 100 gene copies for ≥ 1 species were eliminated. Using the two-lambda (λ) model, the obtained gene families and the ultrametric species tree were used to evaluate the gene family contraction and expansion, where λ indicates a random birth–death parameter. Those with > 10 genes were considered as highly expanded or contracted gene families among the obtained gene families. The resultant highly contracted and expanded gene families were annotated using eggNOG-mapper v2.1.9^127^. Further, the functional annotation of these highly expanded or contracted gene families were check manually on the Kyoto Encyclopaedia of Genes and Genomes (KEGG) database^137^.

### Orthologous gene clustering

The comparative genome-wide identification of orthologous genes across six genomes of Ranunculales order i.e., *Coptis chinensis*, *Corydalis tomentella*, *Kingdonia uniflora*, *Papaver somniferum*, *Thalictrum thalictroides*, and *Tinospora cordifolia* was conducted through OrthoVenn3 (https://orthovenn3.bioinfotoolkits.net/home)^138^. The unique genes of *T. cordifolia* were obtained from this analysis.

### Genes with signatures of adaptive evolution

The above mentioned six Ranunculales species were used for identification of genes with evolutionary signatures. OrthoFinder v2.5.4 was used to construct orthologous gene sets using the proteome files from six selected species^130^. Orthogroups containing protein sequences from all six species were taken further for analysis. In case of more than one protein sequence occurrence for a species, the longest isoform was retained for analysis.

#### Genes with high nucleotide divergence

MAFFT v7.310 was used to individually align the orthogroups across six species^132^. These alignments were used by RAxML v8.2.12 to create orthogroups specific phylogenetic tree with the ‘PROTGAMMAAUTO’ amino acid substitution model and 100 bootstrap value^133^. The root-to-tip branch length distance was calculated using the R package “adephylo” for every gene of all six species in the phylogenetic trees^139^. The genes of *T. cordifolia* with relatively higher root-to-tip branch length distance values were extracted and considered as genes with higher evolution rates or nucleotide divergence.

#### Genes with unique amino acid substitutions with functional impact

Multiple sequence alignments were used to identify the protein sequences with identical amino acid positions in all species except *T. cordifolia*. The gaps and 10 positions around the gaps in the alignments were not considered for this analysis. Sorting Intolerant From Tolerant (SIFT) with UniProt database was used to evaluate the functional impact of extracted sequences with amino acid substitutions. The obtained genes were considered genes with unique amino acid substitutions with functional impact^140^.

#### Positively selected genes

The nucleotide sequences of all orthogroups across selected six species were used by MAFFT v7.310 to obtain individual alignments^132^. The obtained alignments in PHYLIP format along with species phylogenetic tree were used by PAML v4.9a with “codeml” program^134^. PAML v4.9a utilized the branch-site model to identify positively selected genes. Likelihood-ratio tests were performed on these PAML-identified genes and genes with < 0.05 FDR corrected p-values were referred to as positively selected genes of *T. cordifolia*. The identification of positively selected codon sites was performed using Bayes Empirical Bayes (BEB) analysis with > 95% probability criteria for the foreground lineage. These positive selected genes were functionally annotated and assigned to Gene Ontology (GO) categories and KEGG pathways using WebGestalt web server and KAAS v2.1, respectively^126,128^.

#### Genes with all three signatures of adaptive evolution

The three evolutionary signatures of adaptation considered were higher evolution rate, positive selection, and unique amino acid substitutions with functional impact. *T. cordifolia* genes that indicated two of the three evolutionary signatures were suggested as genes with multiple signatures of adaptive evolution (MSA)^118,124,141–143^. The *T. cordifolia* genes showing all three signatures were considered genes with three signatures of adaptive evolution. The eggNOG-mapper v2.1.9 was used to assign KEGG Orthology (KO) and KEGG pathway to the gene sets showing a higher rate of evolution, unique amino acid substitutions with functional impact, positive selection and three signatures of adaptive evolution^127^. The KEGG database was used to retrieve the functional annotation of gene sets from the comparative evolutionary analysis^137^.

### Benzyl iso-quinoline alkaloid (BIA) biosynthesis pathway

The protein sequences of genes involved in BIA biosynthesis for *Papaver somniferum* or *Coptis chinensis* were downloaded from UniProt database^144^. The BIA biosynthetic genes were checked for presence in *T. cordifolia* using blastp^116^. The exon–intron structures of these BIA biosynthetic genes in *T. cordifolia* and other five Ranunculales species were constructed using Exonerate v2.4.0 (https://github.com/nathanweeks/exonerate).

The distant orthologs of 15 genes involved in BIA biosynthesis pathway were identified to understand their origin. The distant orthologs were identified by HHblits web server using UniRef30 database and default parameters^143,145^. The top 20 hits were extracted considering unique genus for each of 15 genes. These 20 hits along with *T. cordifolia* sequence were aligned for each of the 15 genes using MAFFT v.7.310^132^. RAxML v8.2.12 with 100 bootstrap value and ‘PROTGAMMAAUTO’ amino acid substitution model was used to construct the phylogenetic trees for each of the genes using the obtained alignments^133^.

### Other secondary metabolites biosynthesis pathway

The protein sequences of genes involved in terpenoid, lignin (backbone pathway for lignans biosynthesis), and flavonoid biosynthesis pathway for Ranunculales order members were obtained from UniProt database. The obtained genes were searched in *T. cordifolia* using blastp^116^. Among these *T. cordifolia* genes, these genes with signatures of adaptive evolution were identified.

### Analysis of Common symbiosis signalling pathway genes

For CSSP, the genes reported in MacLean et al. (2017) were found in UniProt database and checked for their presence in *T. cordifolia* using blastp^116,144,146^. The genes were manually checked for signatures of adaptive evolution in *T. cordifolia*.

### Analysis of adventitious root formation genes

The genes reported by Li et al. (2021) were obtained from Uniprot database and checked for their presence in *T. cordifolia* using blastp^116,144,147^. The genes were manually checked for signatures of adaptive evolution in *T. cordifolia*.

### Gene expression analysis

Kallisto was used to quantify the transcripts of *T. cordifolia*^148^. The TPM (Transcripts Per Million) values of genes involved in BIA biosynthesis, terpenoid biosynthesis, lignin biosynthesis, flavonoid biosynthesis, CSSP, formation of adventitious root formation, and genes showing all three signatures of adaptive evolution were analyzed.

### Supplementary Information

Supplementary Information 1.

Supplementary Information 2.

## Data Availability

The raw data has been submitted to NCBI database with BioProject accession number PRJNA749156, Biosample accession SAMN20355817, and SRA accessions SRR15221491 and SRR15221490.

## Abbreviations

SARS-COV-2: Severe Acute Respiratory Syndrome Coronavirus 2
COVID-19: Coronavirus disease 19
BLAST: Basic local alignment search tool
ITS: Internal transcribed spacer
MatK: Maturase K
LTR: Long terminal repeat
PEX: Peroxin
CAM: Crassulacean acid metabolism
MYA: Million years ago
CI: Confidence interval
