# Supplementary material for: Genome sequencing and functional analysis of a multipurpose medicinal herb Tinospora cordifolia (Giloy)
Source: Sci Rep. 2024 Feb 2;14:2799. doi: 10.1038/s41598-024-53176-z (PMC10837142; doi:10.1038/s41598-024-53176-z)
Supplement: Supplementary file 1 — Supplementary Information 1. [file 41598_2024_53176_MOESM1_ESM.docx]

**SUPPLEMENTARY INFORMATION**

**Title: Genome sequencing and functional analysis of a multipurpose medicinal herb *Tinospora cordifolia* (Giloy)**

**Authors:** Shruti Mahajan, Abhisek Chakraborty, Manohar S. Bisht, Titas Sil, Vineet K Sharma*

**Affiliation:** MetaBioSys Group, Dept. of Biological Sciences, Indian Institute of Science Education and Research Bhopal, Bhopal, Madhya Pradesh, 462066, India

*Corresponding Author email:

Vineet K Sharma - vineetks@iiserb.ac.in

**E-mail addresses of authors:** Shruti Mahajan - shruti17@iiserb.ac.in, Abhisek Chakraborty - abhisek18@iiserb.ac.in, Manohar S. Bisht – manohar21@iiserb.ac.in, Titas Sil – titassil10@gmail.com, Vineet K Sharma - [vineetks@iiserb.ac.in](mailto:vineetks@iiserb.ac.in)

**SUPPLEMENTARY TEXT: Experimental Methods**

**Sample collection and Nucleic acids Extraction**

The plant was brought from a nursery in Bhopal, Madhya Pradesh, India (23.2599° N, 77.4126° E). The plant was morphologically characterised by its climbing nature, alternatively arranged heart-shaped leaves, and papery bark with the help of Plants of the World Online, Royal Botanic Gardens Kew (<https://powo.science.kew.org/>) and India Biodiversity Portal (<https://indiabiodiversity.org/>). The plant species identity was also confirmed by the sample provider in the nursery. The leaves were collected from the young plant and used to extract nucleic acids (DNA and RNA). The leaves were cleaned and homogenised in liquid nitrogen using a pre-cooled autoclaved mortar-pestle. The young leaves were taken as starting material for nucleic acid extraction as the young leaves may have lesser secondary metabolite content than older leaves[^1^](#_ENREF_1). For DNA extraction, the powdered leaves were washed twice with 70% ethanol and autoclaved distilled water. The washing procedure was done by supplementing distilled water and ethanol with β-mercaptoethanol. The washed homogenate was added to pre-heated Carlson lysis buffer [100 mM Tris-HCL; 2% CTAB; 1.4 M NaCl; 1% PEG 8000; 20 mM Ethylene Diamine Tetra Acetic acid (EDTA)] supplemented with β-mercaptoethanol and polyvinyl pyrrolidone 40 (PVP40). For protein hydrolysis and RNA degradation, 25 µl of Proteinase K (Qiagen, CA, USA) and 2 µl of RNase A (Purelink, Invitrogen, USA) were added and incubated at 65˚C for six hrs at 250 rpm. The tubes were intermittently mixed by inversion during the incubation. After incubation, the tubes were centrifuged at 2500xg for 2 mins at 4˚C, and the supernatant was collected in a new centrifuge tube. The supernatant was treated with 5 µl of RNase A and incubated for 30 mins at 37˚C for complete RNA degradation. After 30 mins, the tubes were added with equal volumes of Chloroform: iso-amyl alcohol (24:1) twice for protein purification. The tubes were mixed by inverting them and centrifuged at 5000xg for 15 mins at 4˚C, and the supernatant was collected in a new centrifuge tube. 0.7x ice-cold isopropanol was added to the supernatant, mixed by inverting and incubated overnight at -20˚C to facilitate DNA precipitation. Following overnight incubation, the tubes were centrifuged at 5000xg for 45 mins at 4˚C. The DNA pellet was washed thrice with 70% ice-cold ethanol. The DNA pellet was air-dried and dissolved in 1 mL of G2 buffer (Qiagen Blood and Cell culture kit). The dissolution was facilitated by incubating them at 50˚C for 15 mins. Till then, the Genomic-tip 20 column was equilibrated using buffer QBT. Four sample tubes were passed through to each equilibrated column so that DNA could bind to the column. The columns were washed with buffer QC thrice. The DNA was eluted in 1 ml of pre-heated QF buffer. Ice-cold isopropanol (0.7x) was added to eluted QF buffer, mixed by inverting the tubes and incubated overnight at -20˚C. The next day, DNA was pellet down by centrifuging at 5000xg for 45 mins at 4˚C. The DNA pellet was washed twice with ice-cold 70% ethanol and air-dried. After adequate drying, the DNA was resuspended in 100 µl nuclease-free water and stored at 4˚C until library preparation. The DNA samples were quantified on Qubit 2.0 fluorometer using a qubit ds DNA BR assay kit (Invitrogen, USA). The DNA samples were checked for quality on Nanodrop 8000 spectrophotometer (ThermoFisherScientific, USA) and proceeded with the library preparation for sequencing. DNA samples with Nanopore-recommended quality values were selected for Nanopore sequencing.

From fresh leaf sample, the total RNA was extracted with TRIzol reagent using multiple reactions. For each reaction, 1 mL of TRIzol reagent (Invitrogen, USA) was added to 100 mg of homogenized leaves and mixed for 5 mins using a vortexer. After adding chloroform (200 µL), the tubes were briefly vortexed and incubated for 10 mins at room temperature. All centrifugation steps were carried out at a speed of 12,000xg and 4˚C temperature. A centrifugation for 15 mins was provided to separate the aqueous layer. The separated aqueous layer was treated with ice-cold isopropanol (500 µL) and incubated for 10 mins at room temperature. A centrifugation for 10 mins was given to pellet down the RNA. The RNA pellet was washed with 1 mL of 70% ethanol, and the residual ethanol was removed by incubating for 30 mins at 37 ˚C. Nuclease-free water (50 µL) was used for resuspending the RNA pellet, followed by an incubation of 10 mins at 55˚C[^2^](#_ENREF_2). The quality and quantity of extracted RNA were evaluated on Nanodrop 8000 Spectrophotometer (ThermoFisherScientific, USA) and 1% agarose gel electrophoresis, and Qubit 2.0 fluorometer with Qubit ssRNA HS assay kit (Invitrogen, USA), respectively. The RNA from multiple reactions was then pooled before proceeding for the library preparation.

**Species Identification**

Along with the morphological identification of the plant mentioned in the previous section, DNA markers were used to further confirm the species identity. Two genes: one nuclear (Internal Transcribed Spacer ITS) and the other plastid gene (Maturase K *matK*), were used to identify the species of the collected sample. The ITS amplification was done using forward primer 5′-TCCGTAGGTGAACCTGCGG-3′ and reverse primer 5′-TCCTCCGCTTATTGATATGC-3′. Similarly, the forward and reverse primers used for *matK* gene amplification were 5’-CGATCTATTCATTCAATATTTC-3’ and 5’-TCTAGCACACGAAAGTCGAAGT-3’, respectively. The PCR programmes used were as follows:

1. For ITS gene amplification:

Initial denaturation: 94 ˚C for 3 mins

35 cycles of –

1. Denaturation at 94 ˚C for 1 min
2. Annealing at 55 ˚C for 1 min
3. Extension at 72 ˚C for 2.5 mins

Final extension: 72 ˚C for 10 mins

1. For *matK* gene amplification:

Initial denaturation: 95 ˚C for 3 mins

35 cycles of –

1. Denaturation at 95 ˚C for 30 sec
2. Annealing at 50 ˚C for 3 mins
3. Extension at 72 ˚C for 1.15 mins

Final extension: 72 ˚C for 7 mins

Taq Polymerase (Invitrogen, USA) and Paq5000 polymerase (Agilent, Santa Clara, USA) were employed for *matK* and ITS amplification, respectively. The amplification was checked by running the amplicons using 2% agarose gel electrophoresis. The amplicons were purified and sequenced on a Sanger sequencer at the in-house facility. The sequences were checked for alignment using blastn with nucleotide database (nt), which showed the highest identity with *Tinospora cordifolia*, confirming the species.

**DNA and RNA Sequencing**

The library for genome sequencing was prepared on a Chromium controller (10x Genomics, CA, USA) using the extracted DNA. The DNA fragments were barcoded and library was prepared on this instrument using Gel bead kit v2 and Chromium Genome Library kit (10x Genomics, CA, USA) as per the manufacturer’s instructions. The library for Nanopore long-read sequencing was prepared using SQK-LSK109 and SQK-LSK110 library preparation kits with a few modifications, like using a starting material of 1.5 µg and increasing the adapter ligation to 30 mins. The library for transcriptome sequencing was prepared using a TruSeq Stranded Total RNA Library Preparation kit with Ribo-Zero Plant workflow (Illumina Inc., CA, USA).

The quality and quantity of both the libraries (10x genomics and transcriptomic) were assessed on Tapestation 4150 (Agilent, Santa Clara, CA) using high sensitive D1000 screentapes and Qubit 4.0 fluorometer using Qubit ds DNA HS assay kit (Invitrogen, USA), respectively. Finally, paired-end reads were generated by sequencing both libraries on NovaSeq 6000 (Illumina, Inc., United States). The prepared Nanopore libraries were loaded on R9.4.1 flowcells and sequenced on MinION sequencers (Mk1B and Mk1C).

**Whole Genome Duplication (WGD) analysis**

*Papaver somniferum* and *Amborella trichopoda* were used to identify Whole Genome Duplication (WGD) event in *T. cordifolia*. The WGD analysis was performed by constructing the whole paranome and orthologs Ks (number of synonymous substitutions per synonymous site) distributions of *T. cordifolia* with *P. somniferum* and *A trichopoda* using wgd v2.0 in default parameters[^3^](#_ENREF_3). The synonymous substitution per site (Ks) distribution of paralogous gene pairs of *T. cordifolia* and *P. somniferum* were found to be ~1.67 and ~ 1.52, respectively **(Supplementary Figure S19)**.

**SUPPLEMENTARY TABLES**

**Supplementary Table S1.** **Detailed information on genomic data for *T. cordifolia***

| **Genomic data** | **Total Number of Reads (Millions)** | **Total Generated Data (Gb)** | **Coverage** |
| --- | --- | --- | --- |
| 10x Genomics (linked reads) | 249,680,248(x2) | 79.4 | ~70x |
| Nanopore reads | 16,000,909 | 22.6 | ~20x |

The sequencing coverage was calculated considering 1.13 Gbp as the estimated genome size

**Supplementary Table S2.** **Description of *T. cordifolia* transcriptome data**

| **Average Read Length R1 (bp)** | **Average Read Length R2 (bp)** | **Total Number of Read pairs** | **Total Number of Bases in R1 (bp)** | **Total Number of Bases in R2 (bp)** | **Total Number of Bases (bp)** |
| --- | --- | --- | --- | --- | --- |
| 161 | 161 | 107,858,957 (x2) | 17,365,292,077 | 17,365,292,077 | 34,730,584,154 |

**Supplementary Table S3.** **Statistical details of final *de novo* assembled draft genome of *T. cordifolia***

| **Parameter** | **Value** |
| --- | --- |
| Number of contigs (>= 1,000 bp) | 55,140 |
| Number of contigs (>= 5,000 bp) | 34,024 |
| Number of contigs (>= 10,000 bp) | 20,185 |
| Number of contigs (>= 25,000 bp) | 10,609 |
| Number of contigs (>= 50,000 bp) | 5,624 |
| Total length (>= 1,000 bp) | 1,014,424,834 |
| Total length (>= 5,000 bp) | 945,658,739 |
| Total length (>= 10,000 bp) | 848,036,722 |
| Total length (>= 25,000 bp) | 699,843,755 |
| Total length (>= 50,000 bp) | 518,106,637 |
| Largest contig | 786,529 |
| GC (%) | 35.11 |
| N50 | 51,235 |
| L50 | 5,409 |
| Number of N's per 100 kbp (>= 2,000 bp) | 402.85 |

**Supplementary Table S4.** **BUSCO assessments of *T. cordifolia* genome**

| **Parameters** | **Assembly Percentage** | **Annotation Percentage** |
| --- | --- | --- |
| Complete BUSCOs (C) | 83% | 60.2% |
| Fragmented BUSCOs (F) | 11.1% | 20.7% |
| Missing BUSCOs (M) | 5.9% | 19.1% |
| Total BUSCO groups searched | 425 | 425 |

The viridiplantae_odb10 database was used as the reference database for BUSCO analysis.

**Supplementary Table S5. Summary statistics of the *T. cordifolia* repetitive genomic regions detected by RepeatMasker**

| Total length: | 1,014,424,834 bp | | | | |
| --- | --- | --- | --- | --- | --- |
| GC (%) | 35.11% | | | | |
| Bases masked: | 768,448,278 bp (75.75 %) | | | | |
|  | | | Number of elements | Length occupied (bp) | Percentage of sequence |
| Retroelements | | | 672,711 | 540,629,624 | 53.29% |
|  | SINEs | | 17,526 | 5,410,294 | 0.53% |
|  | Penelope | | 0 | 0 | 0% |
|  | LINEs | | 98,543 | 44,310,009 | 4.37% |
|  | CRE/SLACS | | 0 | 0 | 0% |
|  | L2/CR1/Rex | | 0 | 0 | 0% |
|  | R1/LOA/Jockey | | 14,014 | 3,182,484 | 0.31% |
|  | R2/R4/NeSL | | 0 | 0 | 0% |
|  | RTE/Bov-B | | 26,521 | 8,103,039 | 0.8% |
|  | L1/CIN4 | | 33,046 | 28,437,389 | 2.80% |
|  | LTR elements | | 556,642 | 490,909,321 | 48.39% |
|  | BEL/Pao | | 6,750 | 1,761,221 | 0.17% |
|  | Ty1/Copia | | 119,769 | 69,409,628 | 6.84% |
|  | Gypsy/DIRS1 | | 403,744 | 400,531,672 | 39.48% |
|  | Retroviral | | 22,119 | 14,656,105 | 1.44% |
| DNA transposons | | | 35,480 | 23,273,632 | 2.29% |
| Rolling circles | | | 42,172 | 10,030,643 | 0.99% |
| Unclassified | | | 457,961 | 153,602,330 | 15.14% |
| Total interspersed repeats | | | | 717,505,586 | 70.73% |
| Small RNA | | | 4,426 | 2,088,531 | 0.21% |
| Satellites | | | 568 | 90,495 | 0.01% |
| Simple repeats | | | 250,381 | 10,413,390 | 1.03% |
| Low complexity | | | 41,363 | 2,024,990 | 0.2% |
|  |  |  |  |  |  |

**Supplementary Table S9.** **Gene families highly contracted in *T. cordifolia***

| **Gene Family ID** | **Description** | **Pathway** |
| --- | --- | --- |
| Gene Family 1 | cytochrome P450, family 94, subfamily C, polypeptide 1 | Cytochrome P450 |
| Gene Family 2 | DnaJ homolog subfamily B member 12 | Protein processing in endoplasmic reticulum |
| Gene Family 3 | hydroxyjasmonate sulfotransferase | Enzymes with EC numbers |
| Gene Family 4 | interleukin-1 receptor-associated kinase 4 | MAPK signaling pathway |
| Gene Family 5 | mTERF domain-containing protein, mitochondrial | Translation factor and Mitochondrial biogenesis |
| Gene Family 6 | omega-hydroxypalmitate O-feruloyl transferase | Cutin, suberine and wax biosynthesis |
| Gene Family 7 | pathogenesis-related protein | Plant-pathogen interaction |
| Gene Family 8 | shikimate O-hydroxycinnamoyltransferase | Phenylpropanoid biosynthesis |
| Gene Family 9 | zinc finger SWIM domain-containing protein 3 | Protein phosphatases and associated proteins |
| Gene Family 10* | Neprosin | - |
| Gene Family 11* | Arabidopsis protein of unknown function | - |
| Gene Family 12* | electron transfer activity | - |
| * Description retrieved from eggNOG mapper | |  |

**Supplementary Table S10.** **Gene families highly expanded in *T. cordifolia***

| **Gene Family ID** | **Description** | **Pathway** |
| --- | --- | --- |
| Gene Family 1 | adenylyl cyclase-associated protein | Membrane trafficking and Exosome |
| Gene Family 2 | adipocyte plasma membrane-associated protein | Membrane trafficking |
| Gene Family 3 | beta-glucosidase | Cyanoamino acid metabolism and Starch and sucrose metabolism |
| Gene Family 4 | caffeic acid 3-O-methyltransferase / acetylserotonin O-methyltransferase | Phenylpropanoid biosynthesis |
| Gene Family 5 | chitinase | Amino sugar and nucleotide sugar metabolism |
| Gene Family 6 | cinnamyl-alcohol dehydrogenase | Phenylpropanoid biosynthesis |
| Gene Family 7 | cytokinin trans-hydroxylase, cytochrome P450 family 709 | Zeatin biosynthesis, Cytochrome P450 |
| Gene Family 8 | interleukin-1 receptor-associated kinase 1 | MAPK signaling pathway |
| Gene Family 9 | interleukin-1 receptor-associated kinase 4 | MAPK signaling pathway |
| Gene Family 10 | male germ cell-associated kinase | Protein kinases and Cilium and associated proteins |
| Gene Family 11 | (R)-mandelonitrile lyase, methyl esterase 5 | Cyanoamino acid metabolism |
| Gene Family 12 | (S)-coclaurine N-methyltransferase-like | Alkaloid biosynthesis |
| Gene Family 13 | pathogen-inducible salicylic acid glucosyltransferase | Tryptophan metabolism and Glucosinolate biosynthesis |
| Gene Family 14 | pectinesterase | Pentose and glucuronate interconversions |
| Gene Family 15 | SAUR family protein | Plant hormone signal transduction |
| Gene Family 16 | soluble epoxide hydrolase / lipid-phosphate phosphatase | Arachidonic acid metabolism |
| Gene Family 17 | transitional endoplasmic reticulum ATPase | Protein processing in endoplasmic reticulum |
| Gene Family 18 | trans-resveratrol di-O-methyltransferase | Stilbenoid, diarylheptanoid and gingerol biosynthesis |
| Gene Family 19 | tRNA-specific adenosine deaminase 2 | Transfer RNA biogenesis |
| Gene Family 20 | ubiquitin C, ubiquitin 13 | Ubiquitin mediated proteolysis |
| Gene Family 21 | UDP-glucosyltransferase 73C | Zeatin biosynthesis |
| Gene Family 22* | lipid-transfer protein DIR1 | Systemic acquired resistance |
| Gene Family 23* | Plant intracellular Ras-group-related LRR protein | Signal Transduction |
| Gene Family 24* | gag-polypeptide of LTR copia-type | Transcription |
| Gene Family 25* | Methyltransferase domain | - |
| * Description retrieved from eggNOG mapper | |  |

**Supplementary Table S17.** **Genes involved in adventitious root formation and showing signatures of adaptive evolution in *T. cordifolia.***

| **Pathway** | **Gene ID** | **Signatures showing** |
| --- | --- | --- |
| Auxin biosynthesis | ASA1 | Positive selection and amino acid substitutions |
|  | ASB1 | Positive selection |
|  | SUR2 | Amino acid substitutions |
|  | GH3-5 | Positive selection and amino acid substitutions |
| Auxin receipt | PIN1 | Positive selection and amino acid substitutions |
| Auxin transport | ABCB19 | Positive selection and amino acid substitutions |
|  | GNOM | Positive selection and amino acid substitutions |
|  | PP2A | High nucleotide divergence |
|  | CML23-1 | Amino acid substitutions |
| Auxin-responsive | ARF17 | Positive selection and amino acid substitutions |
|  | SAUR15 | Highly expanded gene family |
| Meristem and primordium formation | RML1 | Positive selection and amino acid substitutions |
|  | AIL1 | Amino acid substitutions |
|  | PLT1 | Amino acid substitutions |
|  | RPD1 | Positive selection and amino acid substitutions |
|  | MOR1 | Positive selection and amino acid substitutions |
|  | RGD1-3 | Positive selection and amino acid substitutions |
|  | RCH1,2 | Positive selection and amino acid substitutions |
|  | SHR | Amino acid substitutions |
|  | SCR | Amino acid substitutions |
|  | SCL | Positive selection and amino acid substitutions |
|  | WOX4 | Positive selection and amino acid substitutions |
|  | WOX5 | Positive selection and amino acid substitutions |
|  | WOX11 | Positive selection and high nucleotide divergence |
| Cell wall modification | CEP1 | All three signatures of adaptive evolution |
|  | PRP1 | Positive selection and amino acid substitutions |

**Supplementary Table S18. Peroxisome-related genes showing signatures of adaptive evolution in *T. cordifolia***

| **Name** | **Signature of adaptive evolution** |
| --- | --- |
| ATP-binding cassette, subfamily D (ALD), member 3 | Amino acid substitutions |
| Catalase | Amino acid substitutions |
| Solute carrier family 25, member 17 | Positive selection |
| Superoxide dismutase, Fe-Mn family | Amino acid substitutions and High nucleotide divergence |
| Mpv17 | Amino acid substitutions |
| Peroxin-3 | Positive selection and Amino acid substitutions |
| Peroxin-19 | Positive selection |
| Superoxide dismutase, Cu-Zn family | Positive selection and Amino acid substitutions |
| Peroxin-7 | All three signatures of adaptive evolution |
| Peroxin-5 | All three signatures of adaptive evolution |
| Peroxin-14 | All three signatures of adaptive evolution |
| Acyl-CoA oxidase | All three signatures of adaptive evolution |
| Long-chain acyl-CoA synthetase | Positive selection and Amino acid substitutions |
| Sarcosine oxidase / L-pipecolate oxidase | All three signatures of adaptive evolution |

**Supplementary Figures**


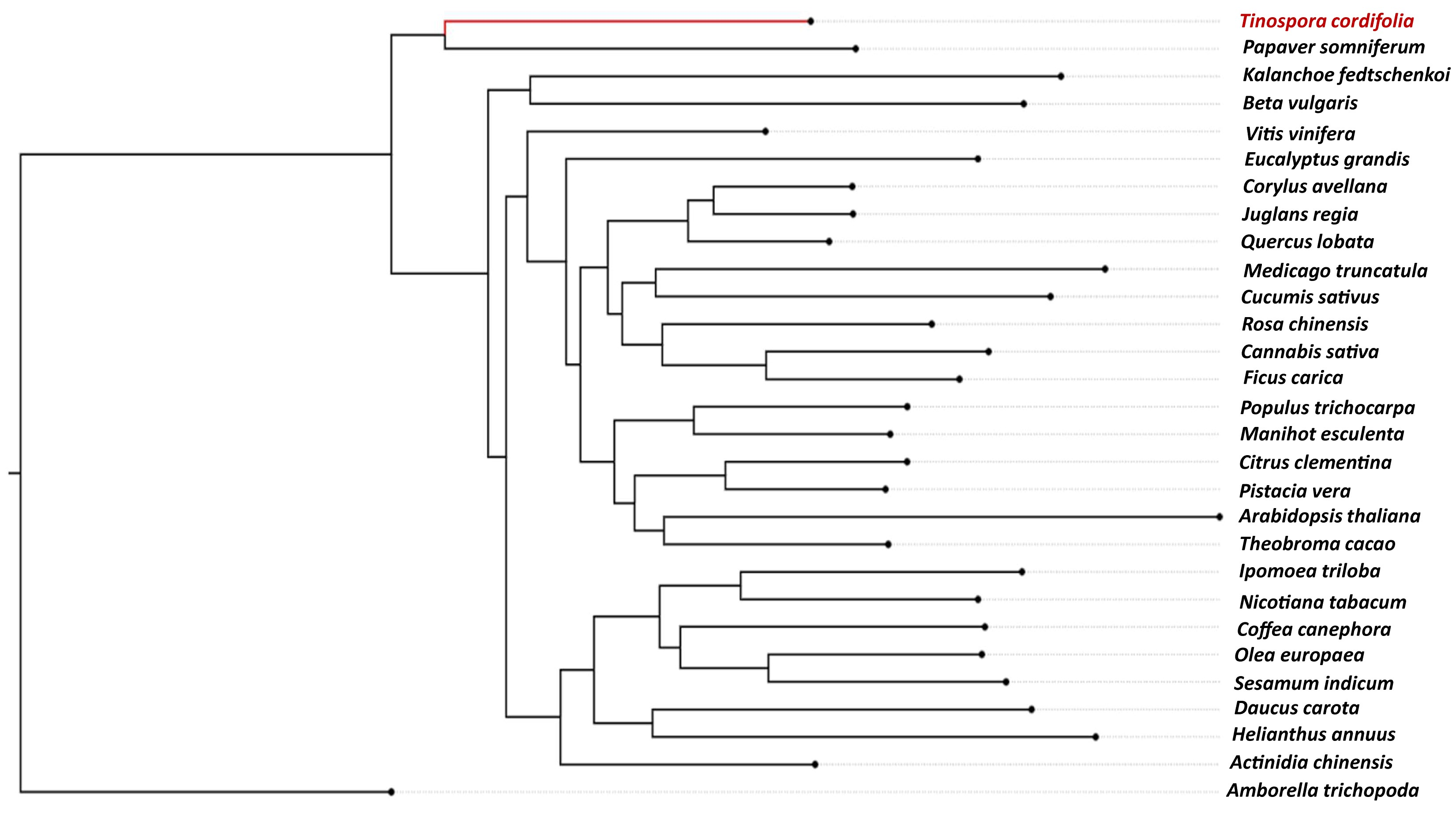


**Supplementary Figure S1. Phylogeny of *T. cordifolia* with 27 eudicot species and *Amborella trichopoda***


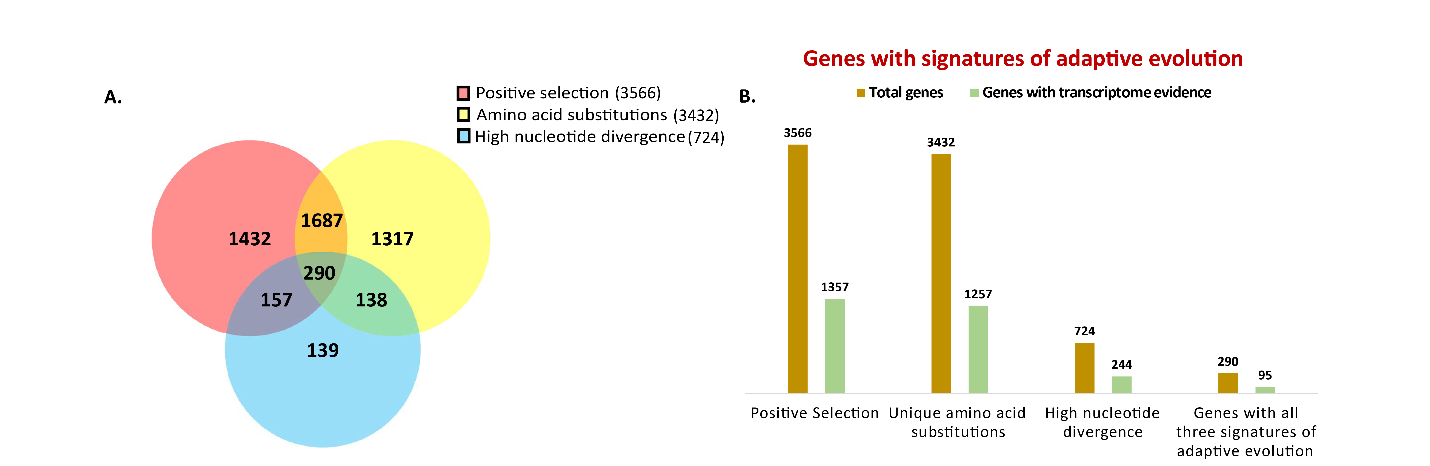


**Supplementary Figure S2. Details on *T. cordifolia* genes with signatures of adaptive evolution A.** Number of *T. cordifolia* genes showing signatures of adaptive evolution **B.** Transcriptomic evidence of genes with evolutionary signatures in *T. cordifolia*.


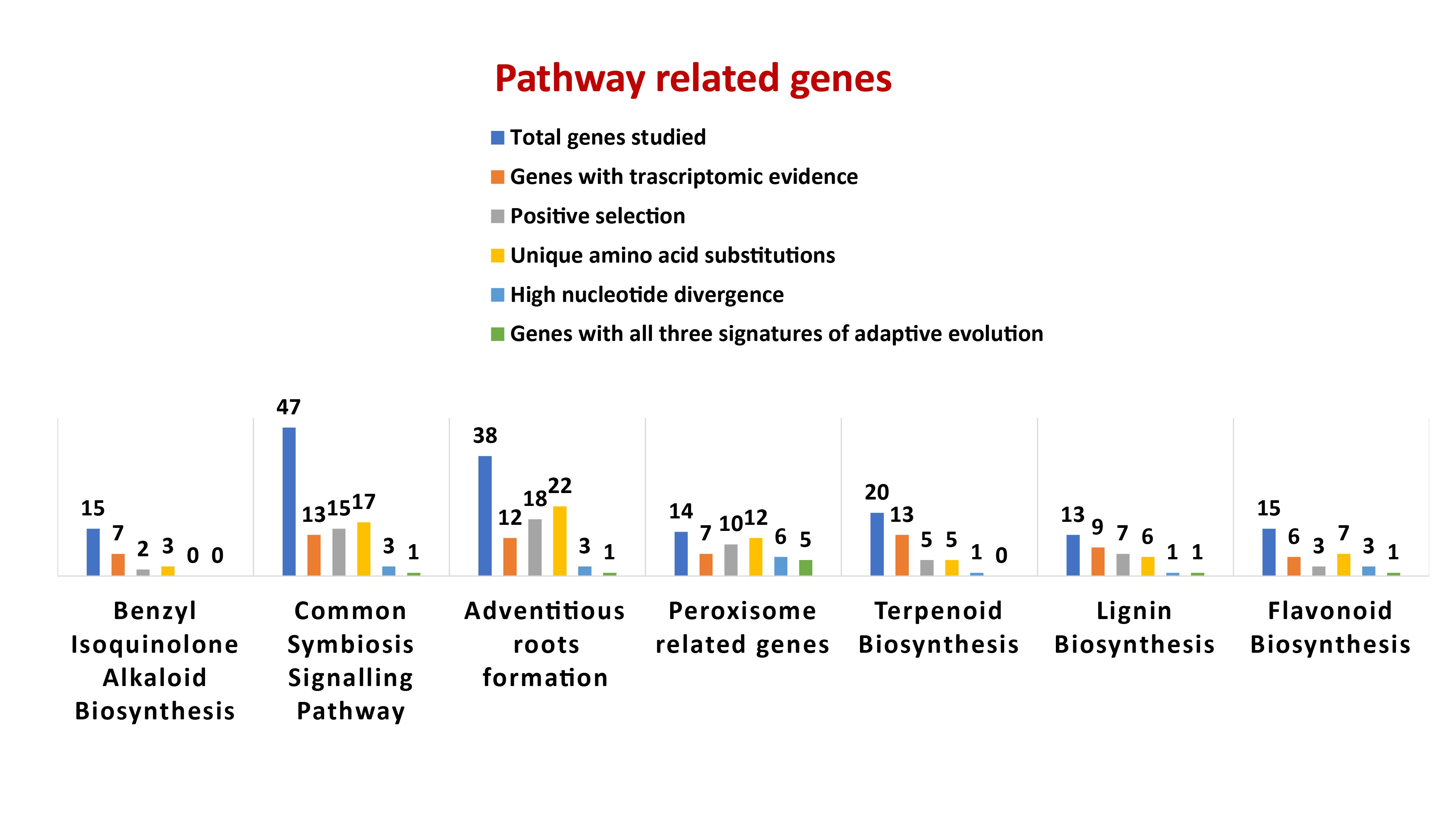
**Supplementary Figure S3. Information on genes involved in various pathways**


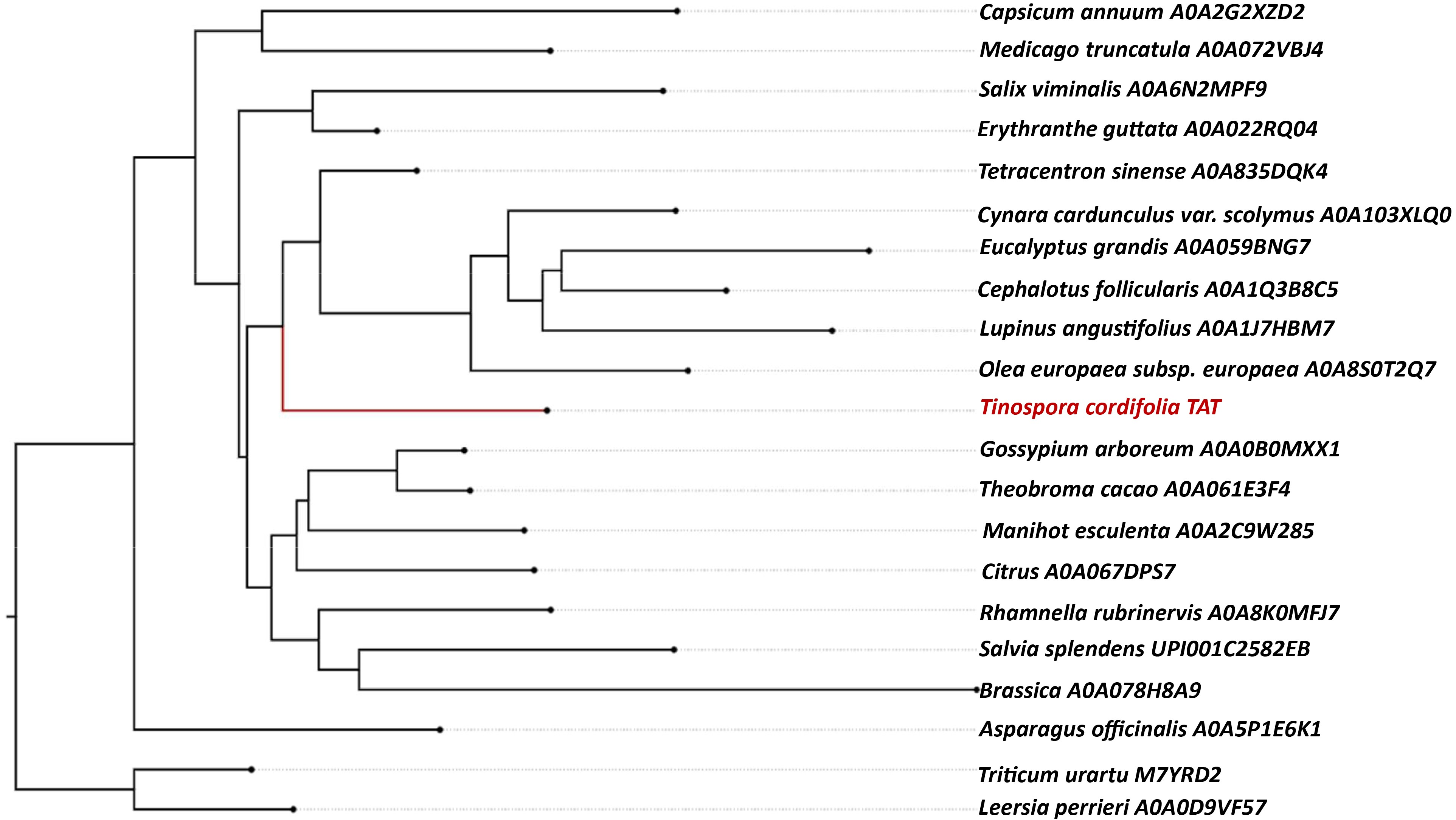


**Supplementary Figure S4. Phylogeny of *T. cordifolia* TAT with top 20 distant orthologs**


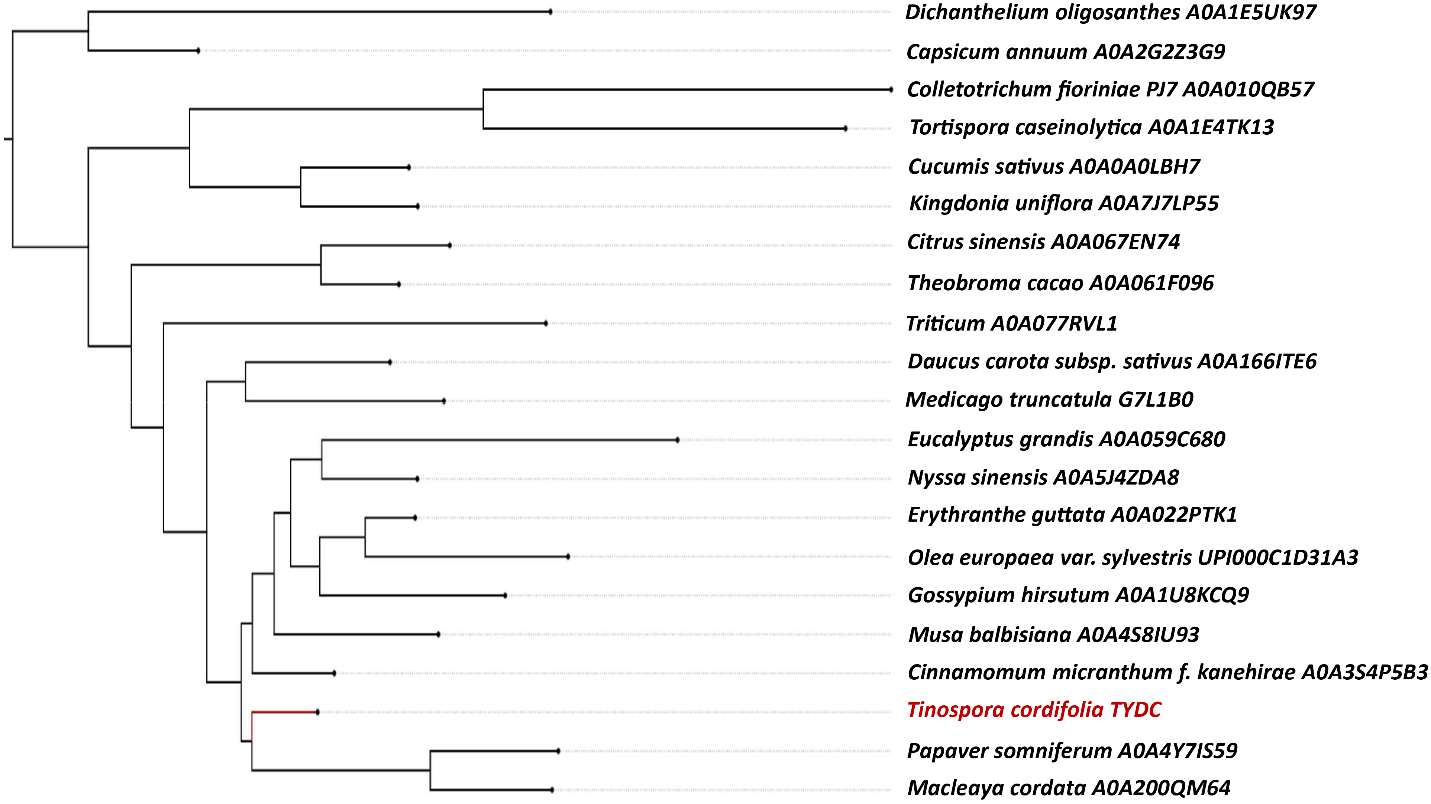


**Supplementary Figure S5. Phylogeny of *T. cordifolia* TYDC with top 20 distant orthologs**


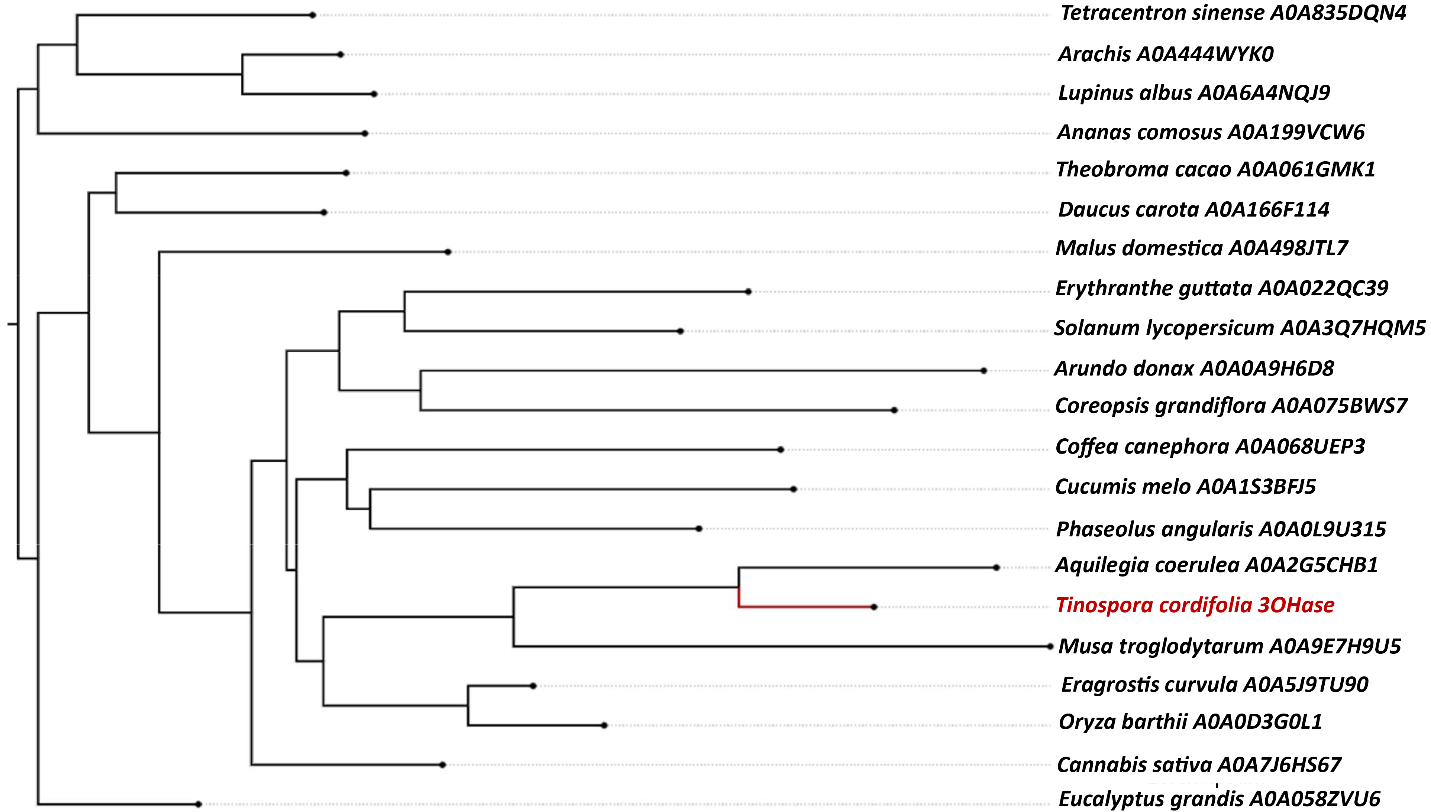


**Supplementary Figure S6. Phylogeny of *T. cordifolia* TYR/3OHase with top 20 distant orthologs**


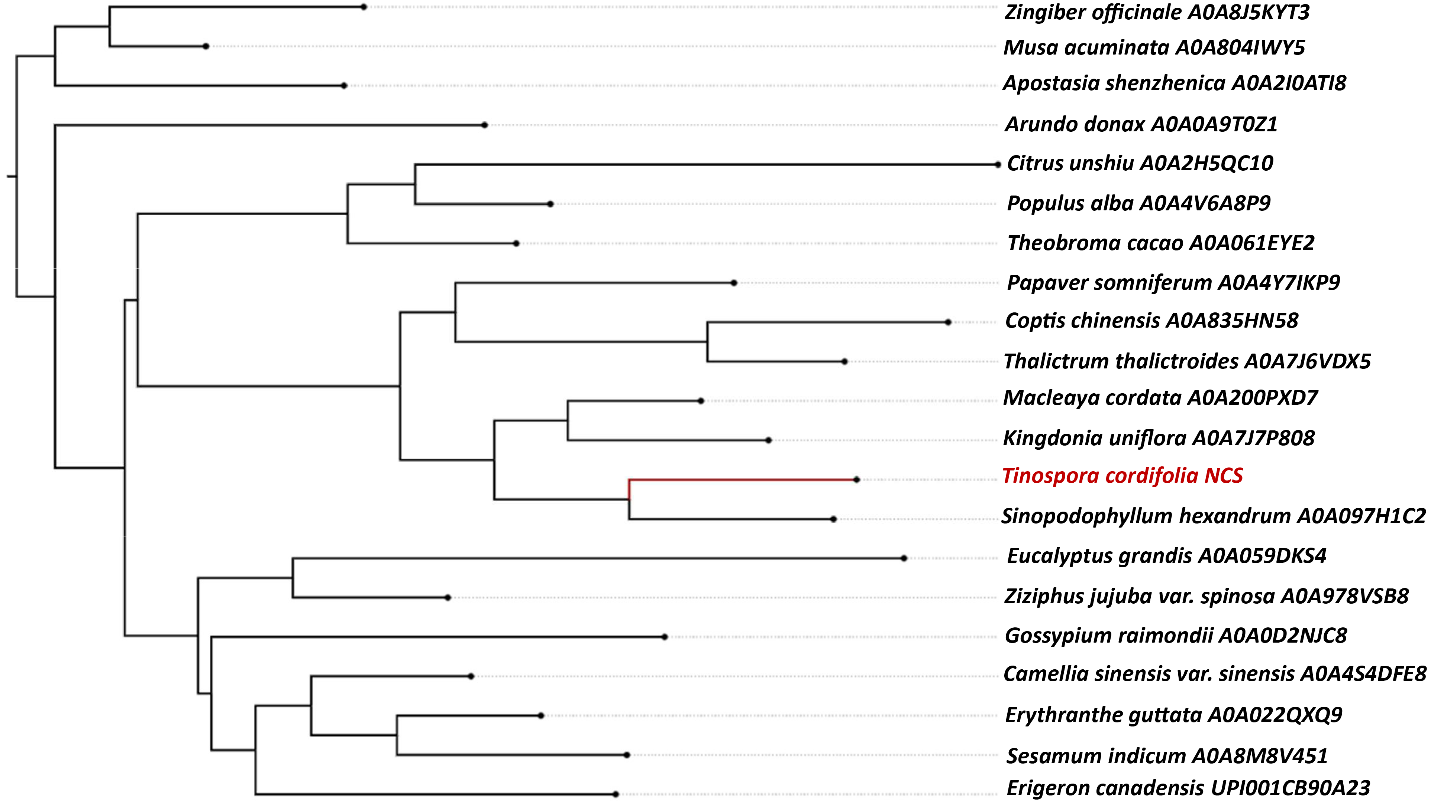


**Supplementary Figure S7. Phylogeny of *T. cordifolia* NCS with top 20 distant orthologs**


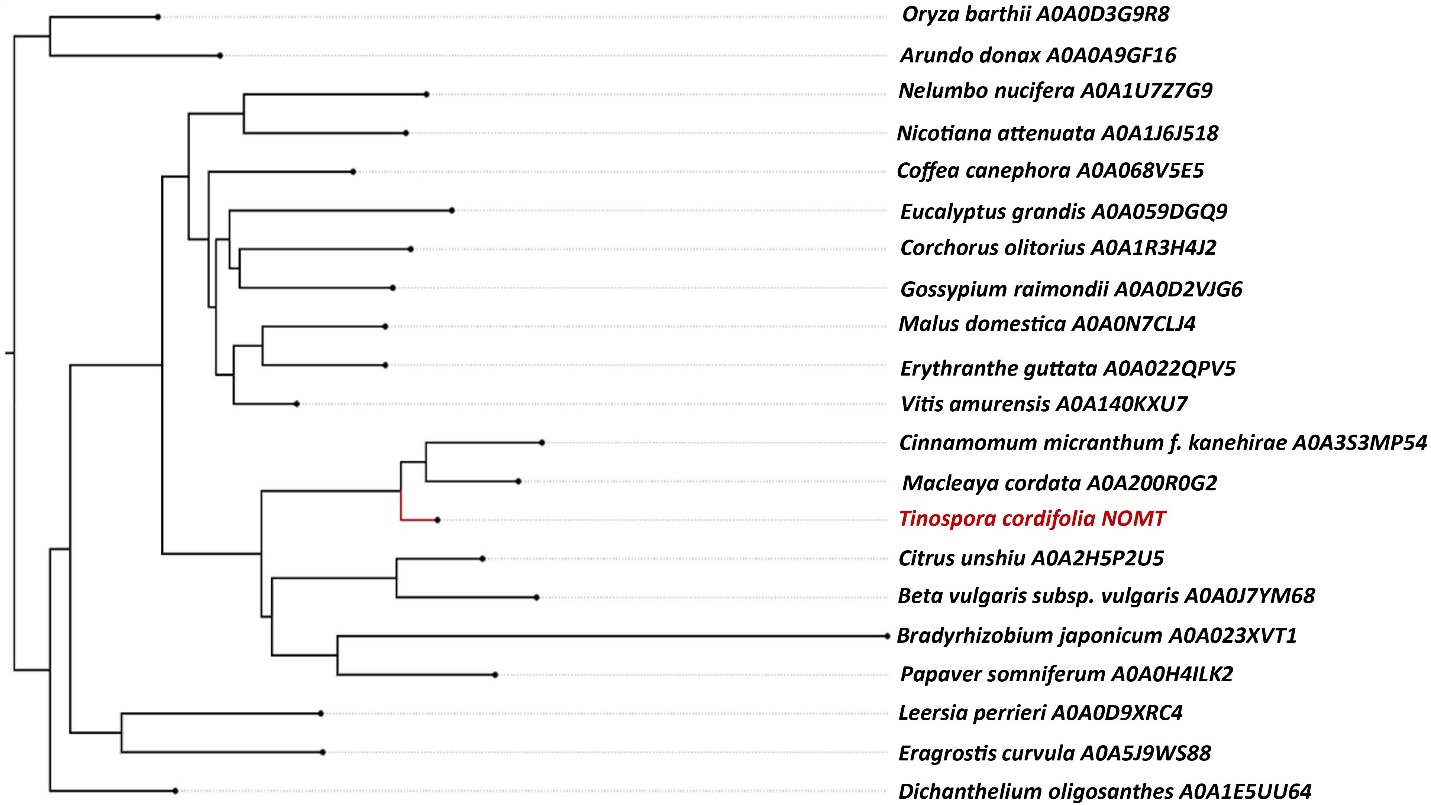


**Supplementary Figure S8. Phylogeny of *T. cordifolia* NOMT with top 20 distant orthologs**


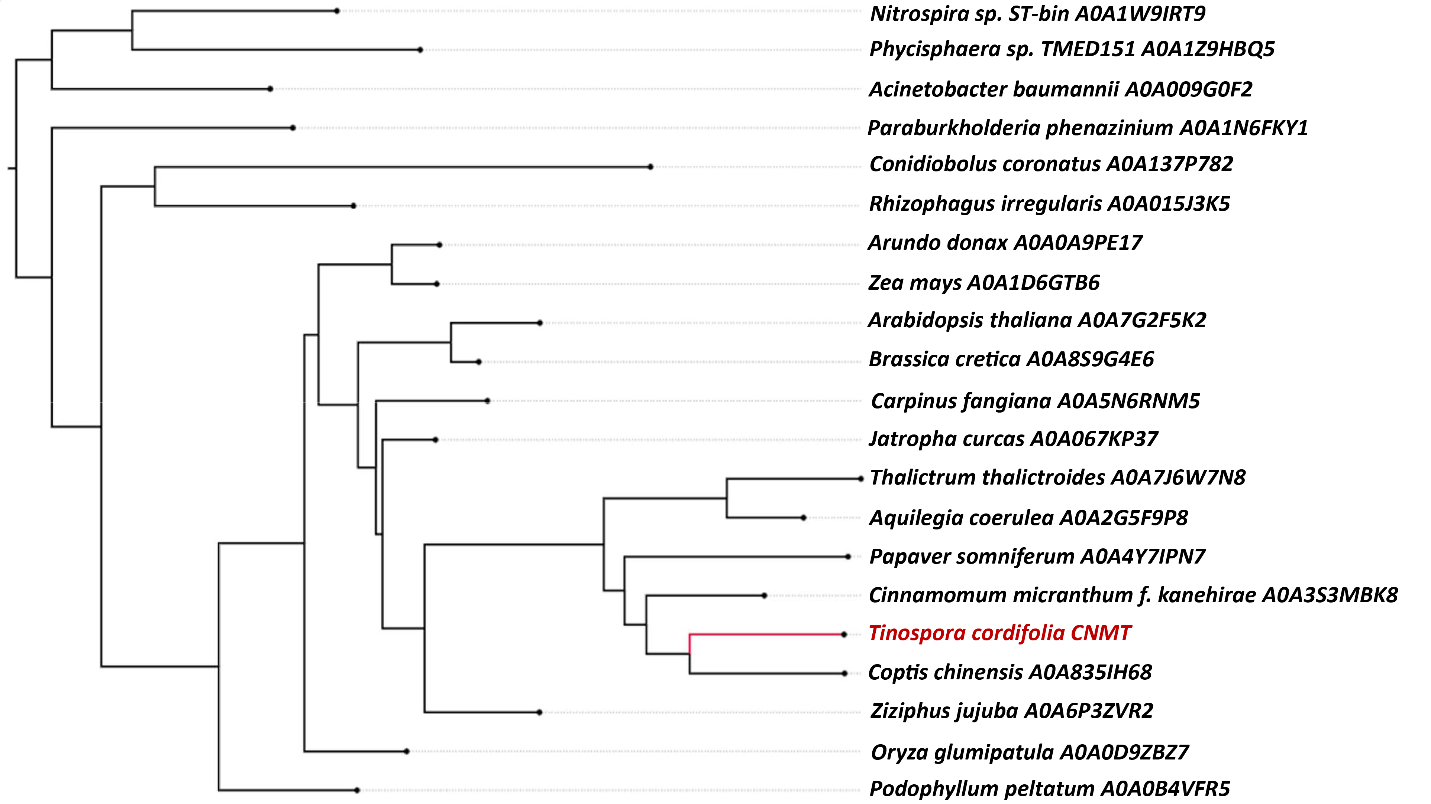


**Supplementary Figure S9. Phylogeny of *T. cordifolia* CNMT with top 20 distant orthologs**


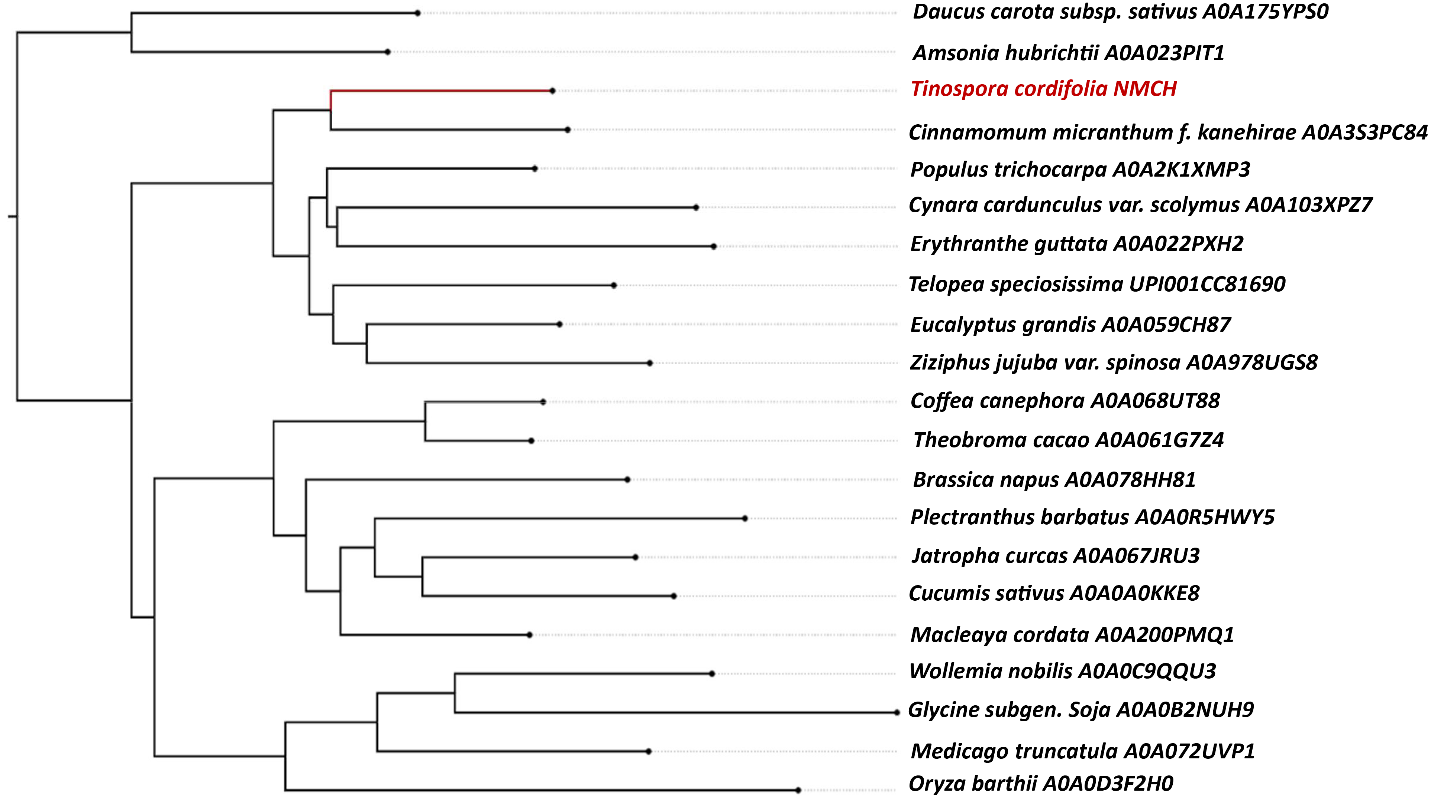


**Supplementary Figure S10. Phylogeny of *T. cordifolia* NMCH with top 20 distant orthologs**


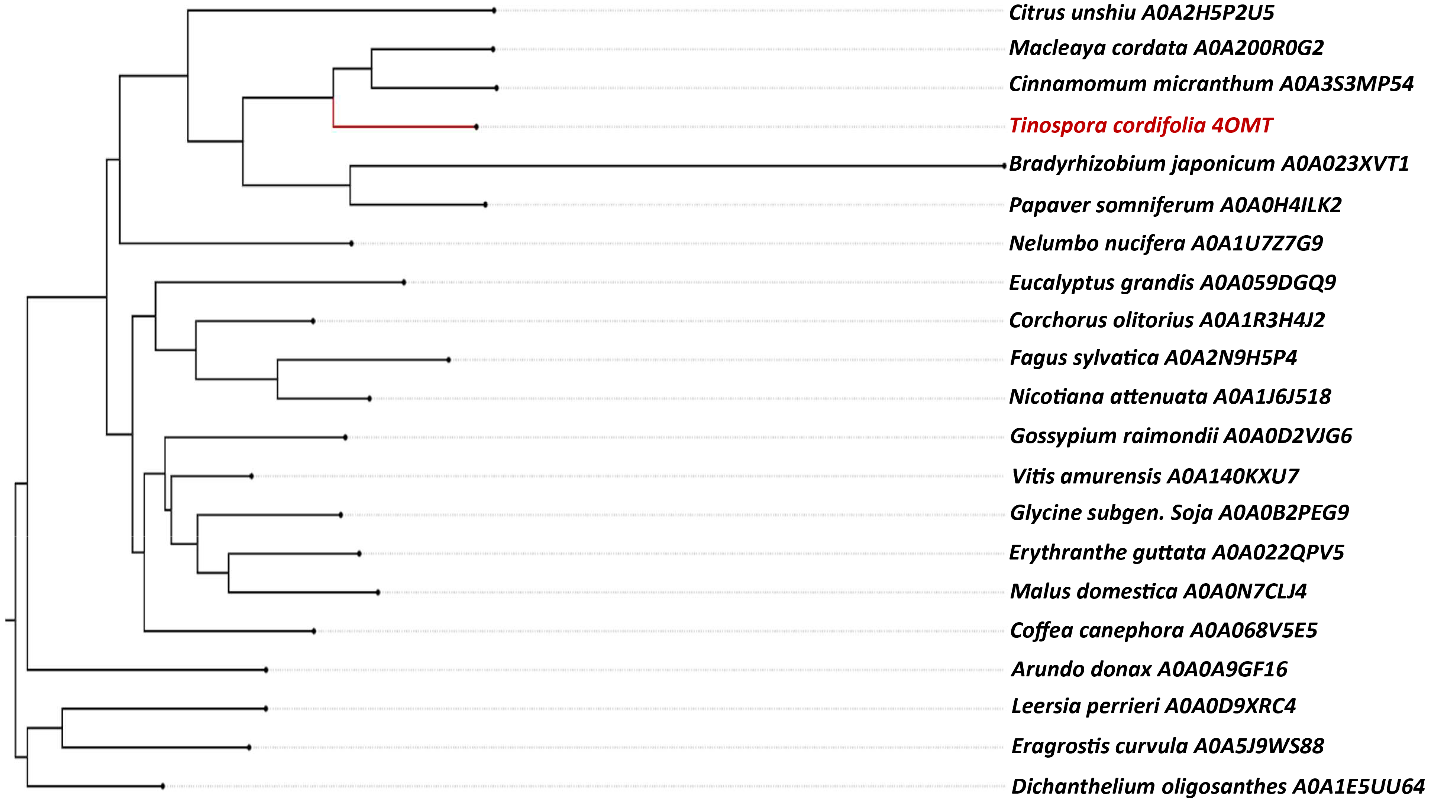


**Supplementary Figure S11. Phylogeny of *T. cordifolia* 4OMT with top 20 distant orthologs**


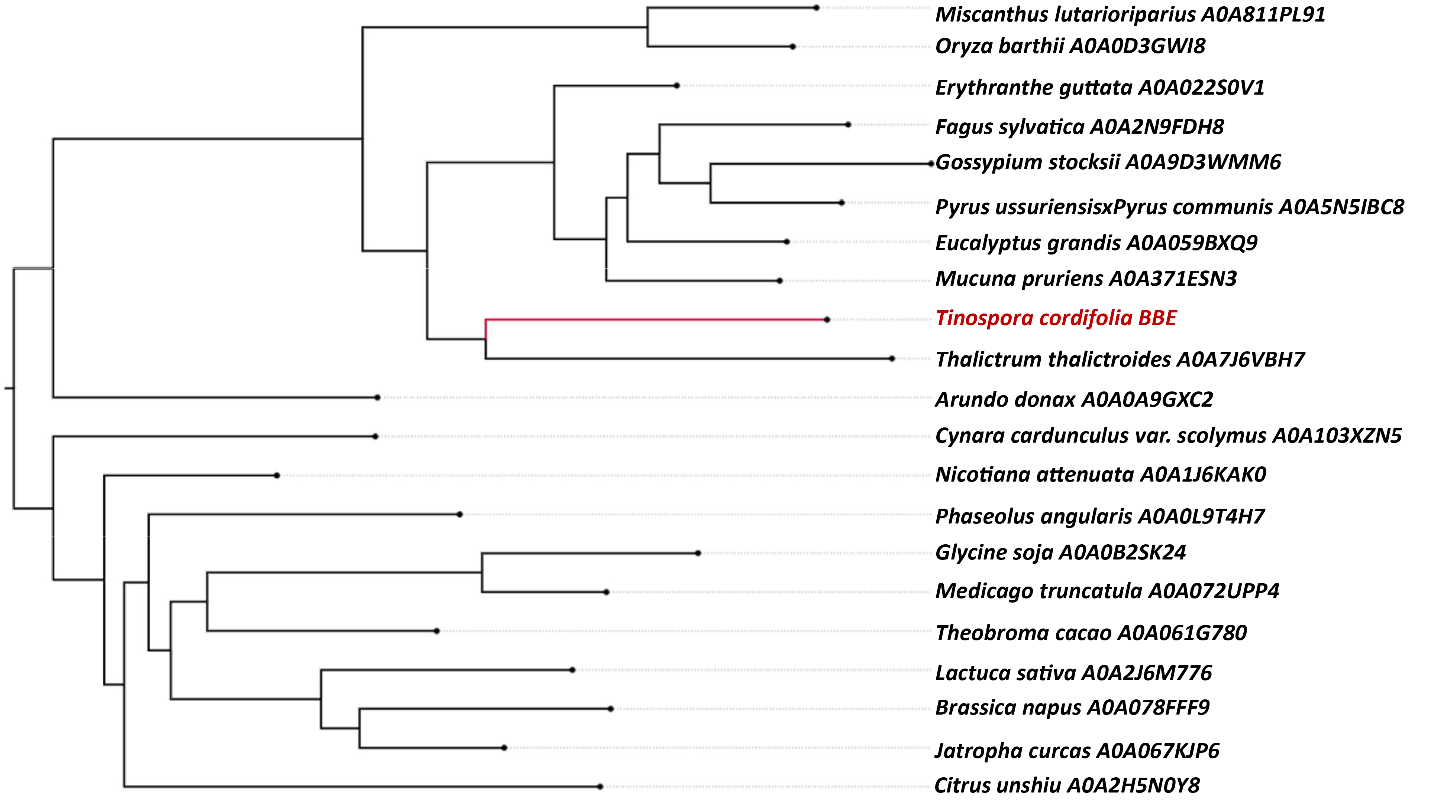


**Supplementary Figure S12. Phylogeny of *T. cordifolia* BBE with top 20 distant orthologs**


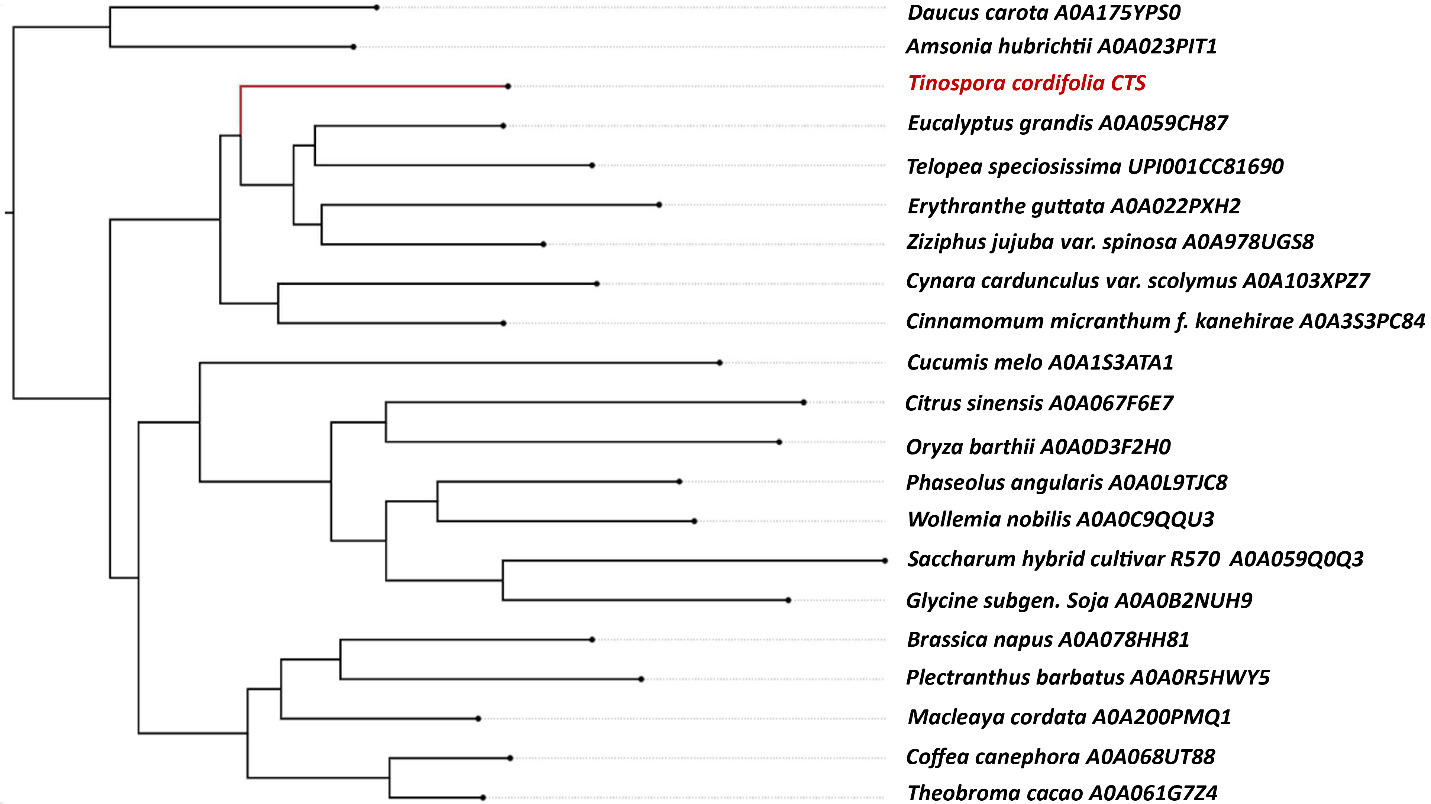


**Supplementary Figure S13. Phylogeny of *T. cordifolia* CTS with top 20 distant orthologs**


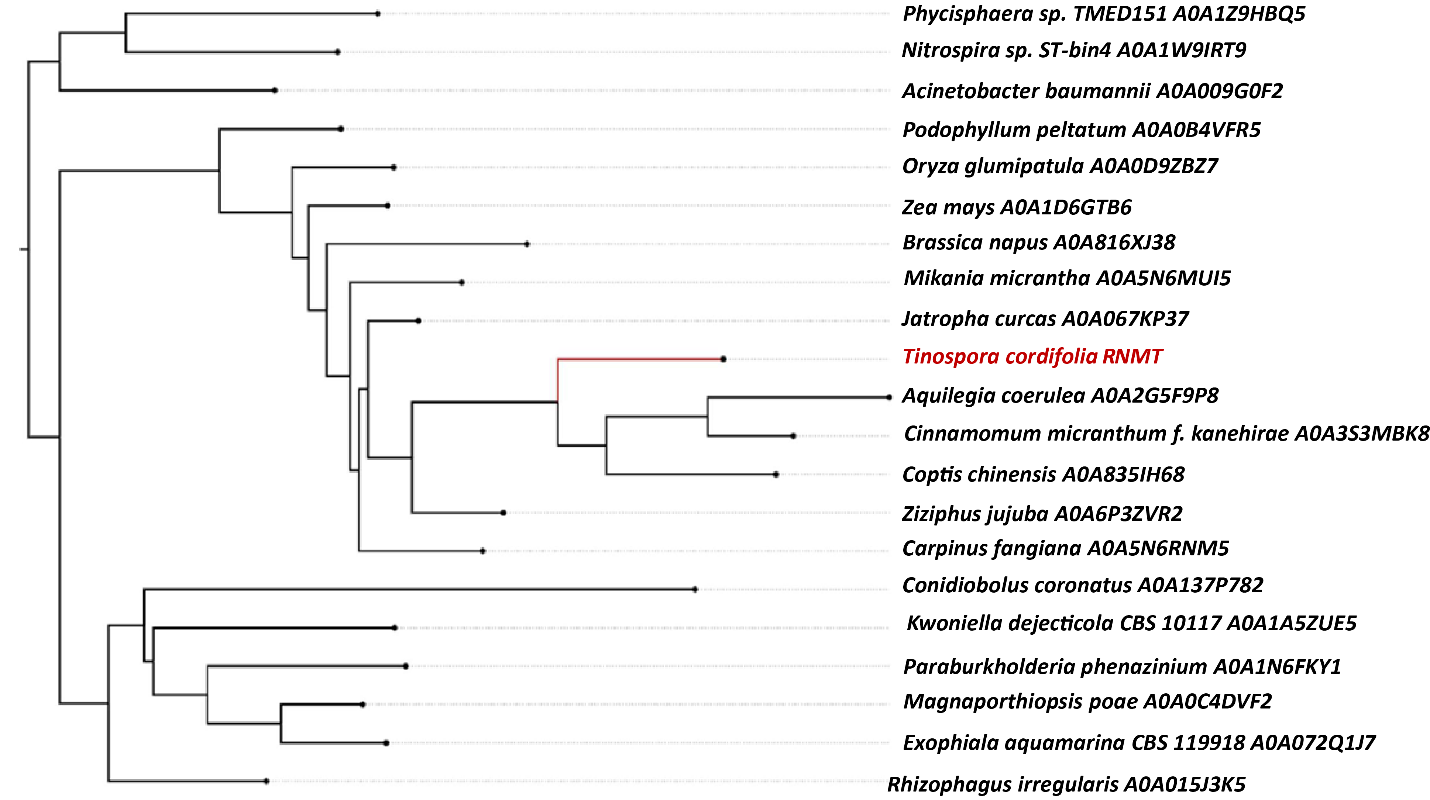


**Supplementary Figure S14. Phylogeny of *T. cordifolia* RNMT with top 20 distant orthologs**


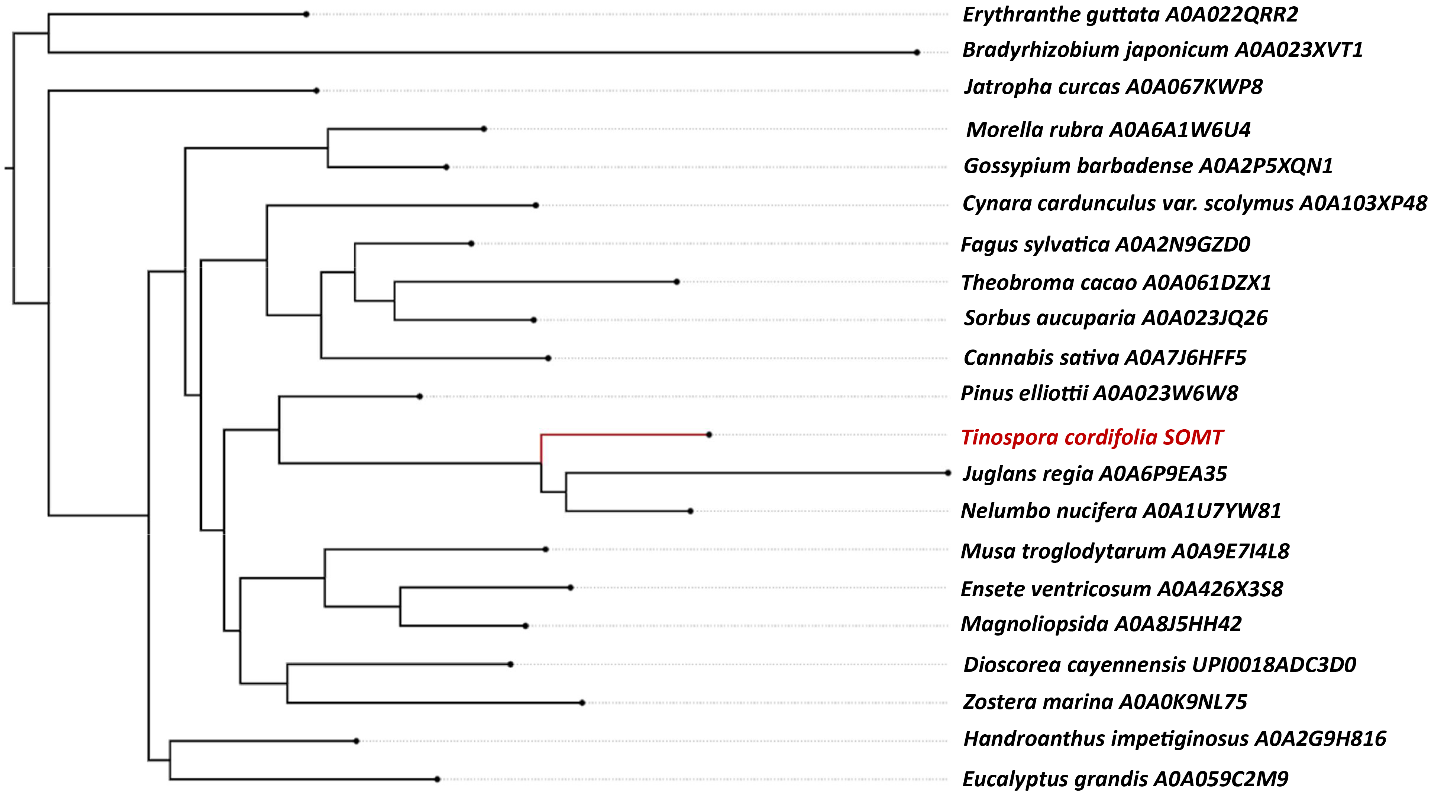


**Supplementary Figure S15. Phylogeny of *T. cordifolia* SOMT with top 20 distant orthologs**


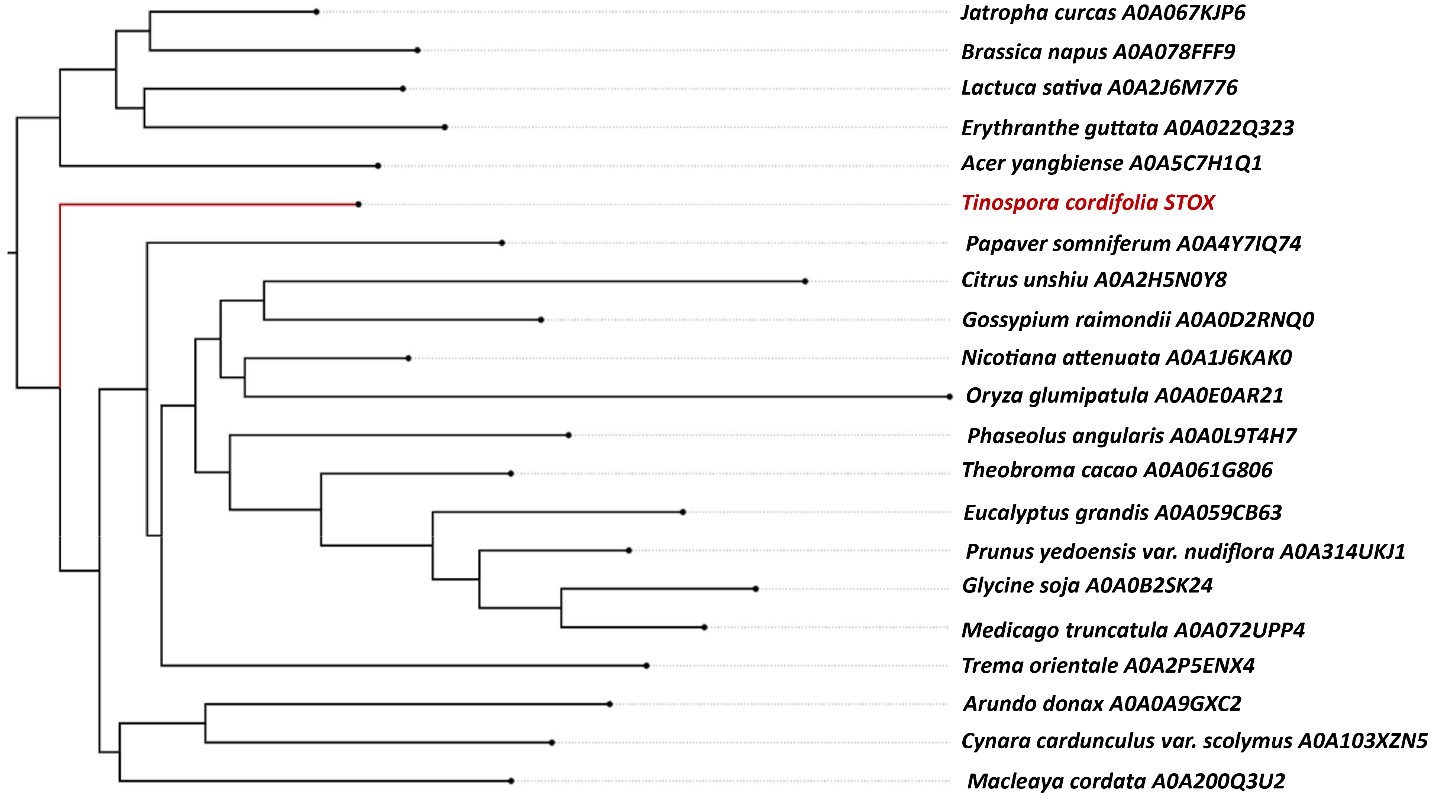


**Supplementary Figure S16. Phylogeny of *T. cordifolia* STOX with top 20 distant orthologs**


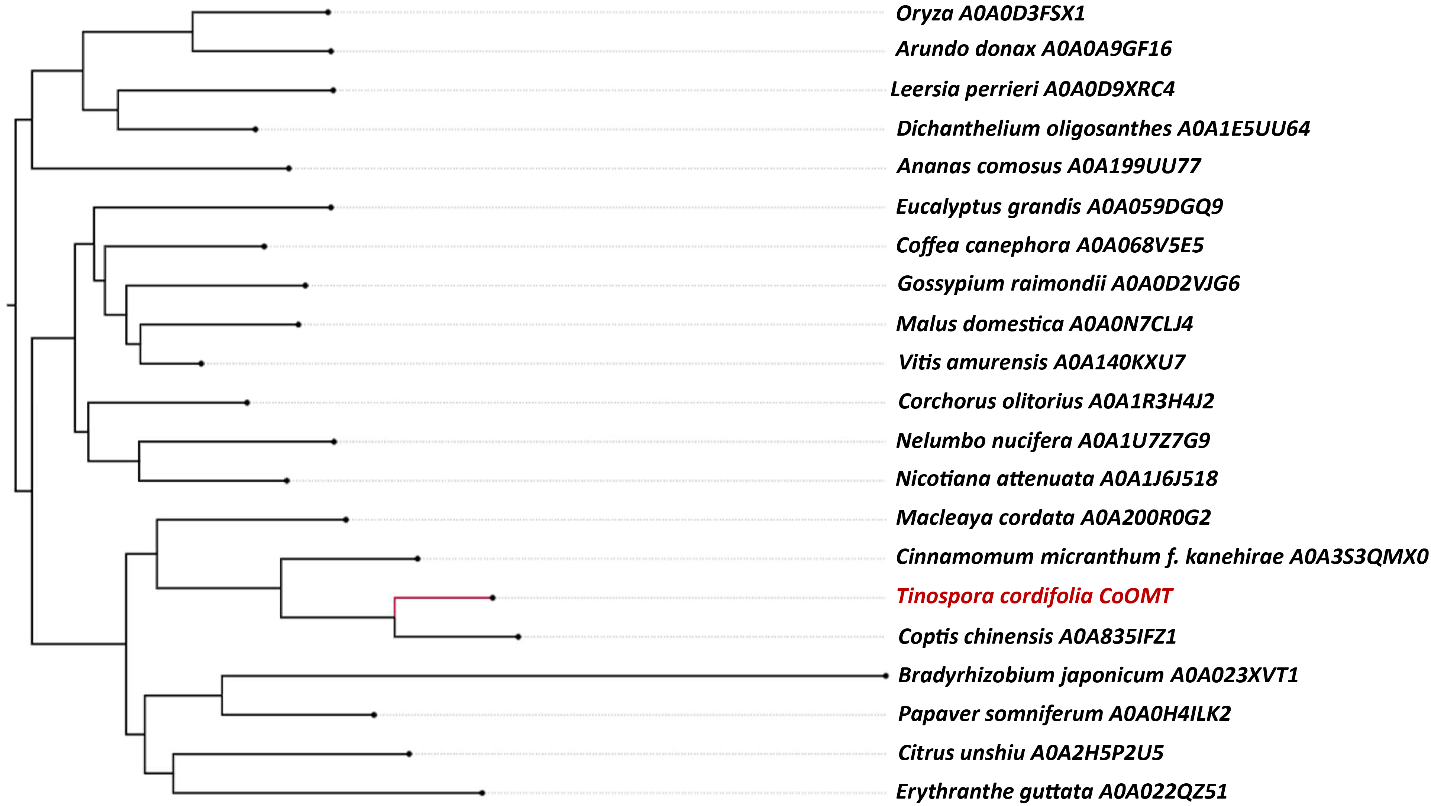


**Supplementary Figure S17. Phylogeny of *T. cordifolia* CoOMT with top 20 distant orthologs**


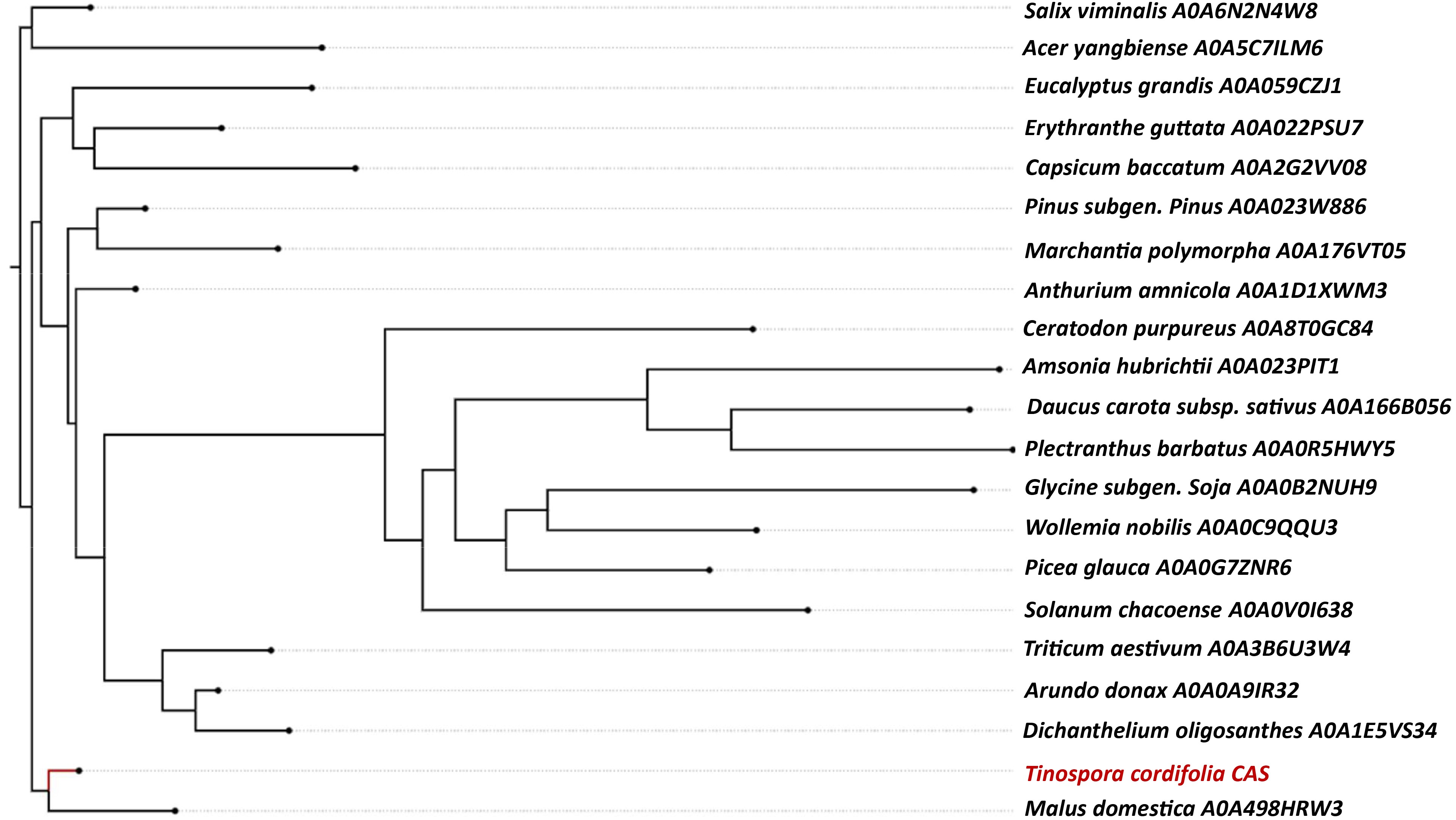
**Supplementary Figure S18. Phylogeny of *T. cordifolia* CAS with top 20 distant orthologs­**


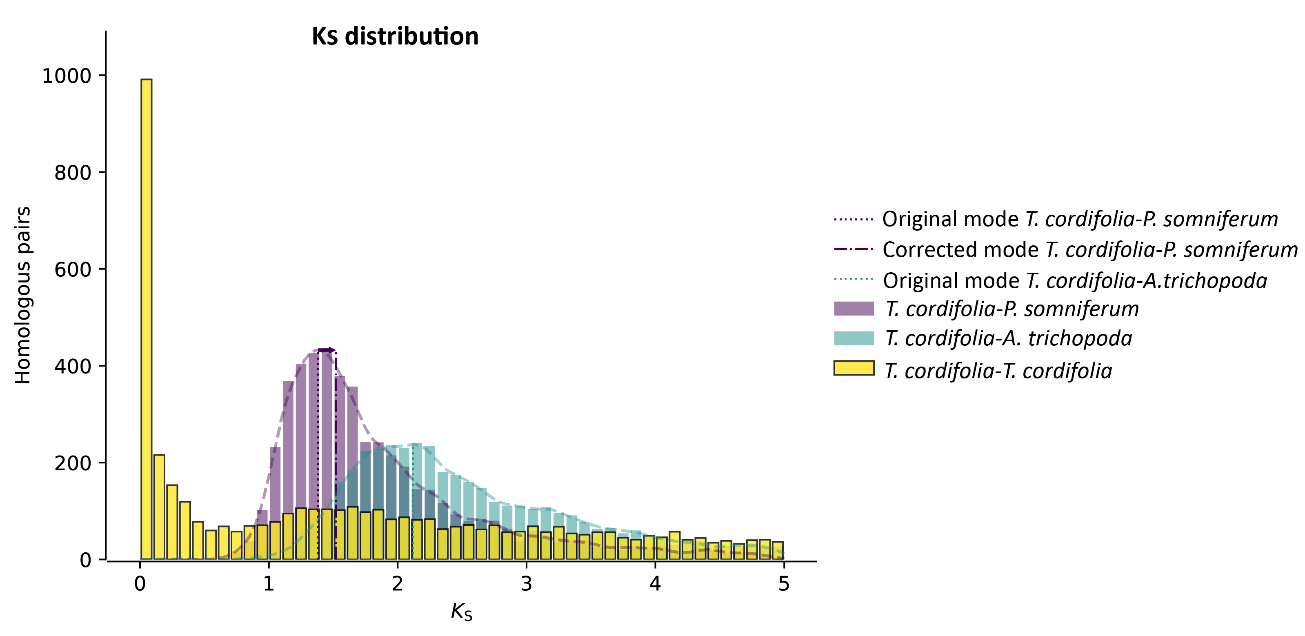


**Supplementary Figure S19. Ks distribution plot for identification of Whole Genome Duplication event in *T. cordifolia***

**REFERENCES**

1. Moreira, P. & Oliveira, D. Leaf age affects the quality of DNA extracted from Dimorphandra mollis (Fabaceae), a tropical tree species from the Cerrado region of Brazil. *Genetics and Molecular Research* **10**, 353-358 (2011).

2. Johnson, M.T. et al. Evaluating methods for isolating total RNA and predicting the success of sequencing phylogenetically diverse plant transcriptomes. *Plos one* **7**, e50226 (2012).

3. Zwaenepoel, A. & Van de Peer, Y. wgd—simple command line tools for the analysis of ancient whole-genome duplications. *Bioinformatics* **35**, 2153-2155 (2019).
